# Basolateral Junction Proteins Regulate Competition for the Follicle Stem Cell Niche in the Drosophila Ovary

**DOI:** 10.1371/journal.pone.0101085

**Published:** 2014-07-03

**Authors:** Maria R. Kronen, Kevin P. Schoenfelder, Allon M. Klein, Todd G. Nystul

**Affiliations:** 1 University of California, San Francisco, Center for Reproductive Sciences, Departments of Anatomy and OB/GYN-RS, San Francisco, California, United States of America; 2 Department of Systems Biology, Harvard Medical School, Boston, Massachusetts, United States of America; University of Bern, Switzerland

## Abstract

Epithelial stem cells are routinely lost or damaged during adult life and must therefore be replaced to maintain homeostasis. Recent studies indicate that stem cell replacement occurs through neutral competition in many types of epithelial tissues, but little is known about the factors that determine competitive outcome. The epithelial follicle stem cells (FSCs) in the Drosophila ovary are regularly lost and replaced during normal homeostasis, and we show that FSC replacement conforms to a model of neutral competition. In addition, we found that FSCs mutant for the basolateral junction genes, *lethal giant larvae* (*lgl*) or *discs large* (*dlg*), undergo a biased competition for niche occupancy characterized by increased invasion of neighboring FSCs and reduced loss. Interestingly, FSCs mutant for a third basolateral junction gene, *scribble* (*scrib*), do not exhibit biased competition, suggesting that Lgl and Dlg regulate niche competition through a Scrib-independent process. Lastly, we found that FSCs have a unique cell polarity characterized by broadly distributed adherens junctions and the lack of a mature apical domain. Collectively, these observations indicate that Lgl and Dlg promote the differentiation of FSC progeny to a state in which they are less prone to invade the neighboring niche. In addition, we demonstrate that the neutral drift model can be adapted to quantify non-neutral behavior of mutant clones.

## Introduction

Adult stem cells are maintained within specialized microenvironments, or niches, that promote stem cell self-renewal while allowing non-stem cells to exit the niche and differentiate [1], [2]. This system ensures that both the stem cell and the differentiated cell populations are replenished during normal homeostasis. However, stem cells can also die or exit the niche, and must be replaced in order to maintain a healthy stem cell pool. Different causes of stem cell loss have been proposed, such as aging, damage, or chance loss of stem cells, but the process is not fully understood.

In many tissues, wildtype stem cell replacement is stochastic, neutral, and can be very frequent, suggesting that it is a normal part of homeostasis that reflects a robust design of tissue stem cell systems. Both this process and the genetic basis for it have been studied extensively in Drosophila, and, more recently, it has become clear that stem cells also regularly turn over in mammalian epithelia. This pattern of stem cell loss and replacement conforms to a model of neutral competition for niche occupancy, which predicts characteristic fluctuations in the size of stem cell clones that are analogous to the neutral drift of alleles in population genetics [3]–[5]. The model relies the notion that each stem cell division produces either a symmetric outcome, which results in two stem cells or stem cell loss through differentiation, or an asymmetric outcome, which results in one stem cell and one daughter cell. The ratio of symmetric to asymmetric outcomes varies between tissue types, but in each tissue, the frequencies of symmetric division and stem cell loss must be equal in order to maintain a constant number of stem cells.

Lineage tracing studies in Drosophila have shown many examples of stem cells that undergo replacement during normal homeostasis [6]–[10]. In addition, genetic mosaic studies have demonstrated robust genetic regulation of stem cell self-renewal [1]. Collectively, these studies show that mutations that disrupt the ability of the stem cell to receive niche signals or adhere to the niche typically cause the mutant stem cells to be lost [11], [12], while mutations that block differentiation or increase adhesion to the niche promote an expansion of the mutant stem cell population [13]–[15]. In these cases, because the loss or gain of mutant stem cells is balanced by a compensatory expansion or contraction of the wildtype stem cell population, the mutant stem cells undergo non-neutral, or biased, competition for the niche.

**Figure 1:**
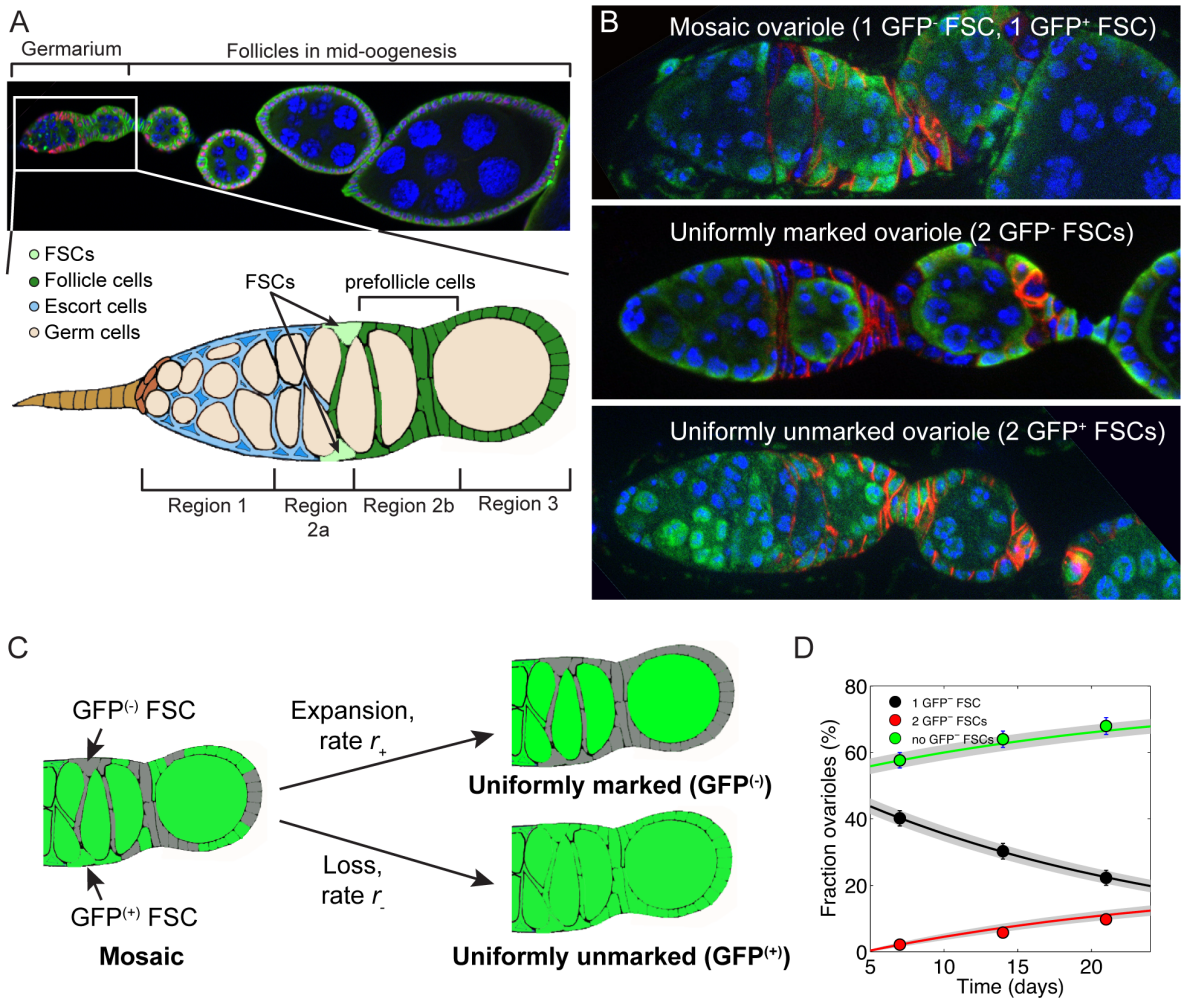
Follicle stem cells undergo neutral drift. **A**. A Drosophila ovariole stained for Hu li tai shao (green) to highlight cell membranes, Traffic jam (red) to highlight somatic cell nuclei and DAPI (blue) to highlight DNA. In each ovariole, new follicles are produced by the germarium (boxed region, diagramed below) at the anterior tip. Follicles move toward the posterior as they mature. The germarium is divided into four sections as indicated, and the FSCs reside at the Region 2a/2b border. Anterior is to the left. **B**. Mosaic, uniformly marked (GFP^-^), and uniformly unmarked (GFP^+^) wildtype ovarioles stained for FasIII (red) to label follicle cells, GFP (green) to mark FSC clones, and DAPI (blue). **C**. Mosaic ovarioles become uniformly marked when the GFP^+^ FSC is replaced by a daughter of the GFP^-^ FSC, or uniformly unmarked when the GFP^-^ replaced by a daughter of the GFP^+^ FSC. These events are referred to as clone expansion (r_+_) or clone loss (r_-_), respectively. **D**. An alignment of the observed fraction of ovarioles with 0, 1 or 2 GFP^-^ FSCs at 7, 14 and 21 dphs with the values predicted by the neutral competition model. The points indicate the actual data, and the error bars indicate the S.E. The solid lines indicate the values predicted by the model and the grey shaded areas indicate the 95% confidence interval ranges.

In this study, we used the follicle stem cells (FSCs) in the Drosophila ovary to investigate the role that cell polarity proteins play in regulating competition for the niche in an epithelial tissue. The FSCs are a highly tractable model of in vivo stem cell biology [16]. Each functional unit of the ovary, called an ovariole, is composed of a germarium followed by a long chain of developing follicles. Clonal analyses using single and multi-color labeling systems have indicated that exactly two active FSCs reside within each germarium at the anterior edge of the follicle epithelium (Fig. 1A) [6], [17]. In well-fed flies, each germarium produces approximately two egg chambers per day [18], and each FSC produces, on average, one daughter cell per egg chamber [19], implying that FSCs divide approximately once every 12 hours. Newly produced FSC daughter cells divide at a slightly faster rate (9.6 hours [6]) and approximately 50% of these cells migrate across the width of the germarium toward the FSC niche on the opposite side [17]. FSCs are occasionally lost from the niche during adulthood and replaced by a cross-migrating daughter [6], and several mutations have been identified that cause an increase or decrease the rate of FSC replacement [20], [21].

Here we show that the loss and replacement of wildtype FSCs occurs seemingly at random, leading to neutral competition for niche occupancy. In addition, we show that FSCs have a distinct, immature cell polarity, and that FSCs that are mutant for either *discs large* (*dlg*) or *lethal giant larvae* (*lgl*), two essential cell polarity genes, undergo biased competition for niche occupancy. In both cases, mutant FSCs show increased competitive behavior. These results suggest that establishment of cell polarity is an important early differentiation event of FSCs, and that daughter cells that maintain an immature polarity state are at an advantage in occupying the niche.

## Results

### FSCs undergo neutral competition

To monitor FSC replacement, we used the GFP-negative Flp/FRT clonal system in which heat shock-induced expression of flippase (Flp) catalyzes FRT recombination and the formation of GFP^-^ wildtype (Fig. 1B) or mutant clones [22]. We induced sparse GFP^-^ FSC clones in adult flies, and examined the clone frequencies at 7, 14, and 21 days post heat shock (dphs). If FSCs undergo neutral competition, the overall frequency of FSCs marked by the lack of GFP should remain constant over time, even as individual stem cells are lost and replaced. Indeed, we found that the frequency of GFP^-^ FSCs was similar at all three time points (22±1.9%, n = 458; 21±2.1%, n = 380; and 21±2.2%, n = 337; at 7, 14, and 21 dphs, respectively, Table 1).

**Table 1:** Quantification of marked FSC clone frequency at 7, 14 and 21 days post heat shock.

	7 days post heat shock			14 days post heat shock			21 days post heat shock			Change from 7 to 21 days post heat shock		
Genotype	1 GFP^-^ FSC	2 GFP^-^FSCs	Total GFP^-^FSCs (n)	1 GFP^-^ FSC	2 GFP^-^FSCs	Total GFP^-^FSCs (n)	1 GFP^-^FSC	2 GFP^-^FSCs	Total GFP^-^FSCs (n)	1 GFP^-^ FSC	2 GFP^-^FSCs	2 GFP^+^ FSCs
Wildtype	40%	2%	22% (458)	30%	6%	21% (380)	22%	10%	21% (337)	−18%	+8%	+10%
lgl(1)	39%	4%	24% (183)	24%	19%	31% (283)	14%	27%	34% (169)	−25%	+23%	+2%
dlg(m52)	33%	3%	20% (394)	19%	17%	26% (322)	11%	21%	27% (200)	−22%	+18%	+4%
scrib(1)	30%	4%	18% (138)	28%	4%	18% (195)	27%	5%	18% (128)	−3%	+1%	+2%
scrib(2)	40%	2%	22% (435)	26%	8%	20% (199)	23%	9%	20% (362)	−17%	+7%	+10%
baz(4)	37%	3%	21% (263)	28%	8%	22% (391)	20%	12%	22% (506)	−17%	+9%	+8%

To measure the rate of FSC replacement, we quantified the proportion of ovarioles with 0, 1, or 2 GFP^-^ FSCs at each time point. Ovarioles that start out as a mosaic, with one GFP^-^ FSC and one GFP^+^ FSC, become fixed in a uniformly marked or unmarked state when one of the FSCs is replaced by a daughter cell of the other (Fig. 1B). Thus, the rate at which mosaic ovarioles decrease in frequency over time can be used to derive the rate of FSC replacement. In addition, the difference between the frequencies of uniformly marked versus uniformly unmarked ovarioles at each time point can be used to measure competition bias. Specifically, if competition is neutral, the frequencies of uniformly marked and unmarked ovarioles will increase at equal rates, whereas if competition is biased, one population will expand at the expense of the other. We found that, in wildtype organisms, the frequency of mosaic ovarioles decreased by 18% from 7 to 21 dphs, and the frequencies of uniformly marked and unmarked ovarioles increased by roughly equal amounts (8±1.4% and 10±1.5%, respectively, Table 1) over this same time period.

To formally evaluate this data and other data that follow later, we made use of a population dynamics model for FSC competition in the follicle epithelium. In general, the patterns of stem cell clone expansion and contraction that occur during neutral competition can be described by the Moran Process and related models [4], [23], which were initially developed for studying genetic drift within isolated populations. Because each ovariole in the Drosophila ovary is a self-contained unit of tissue with precisely two active FSCs [6], [17], we adapted these models to consider competition between just two stem cells, using a similar framework to that applied in other stem cell systems [4]. In this model (see Methods), we assumed that each FSC may be lost in a stochastic event, after which it can be replaced by its own progeny, or by invasion from the neighboring FSC. After clone induction, if the marked FSC is lost, the GFP^-^ clone vanishes. Conversely, if the marked FSC invades the neighboring niche, the GFP^-^ clone expands and becomes fixed in the ovariole (Fig. 1C). The stochastic nature of FSC loss or replacement should lead to an exponential decay in the fraction of ovarioles containing just a single marked FSC, with the decay constant corresponding to twice the average FSC lifetime (Methods). We indeed observed this characteristic exponential decay in wildtype FSCs (Fig 1D, *r* ^2^>0.99). This observation further indicates that the rate of FSC replacement did not change with age, remaining constant throughout the entire period of observation.

Using the FSC competition model, we defined an “FSC competition bias”, *b*, as the relative difference between the rates of FSC loss and FSC expansion (see Methods). A bias of *b* = 0 indicates neutral competition, a positive bias (up to 100%) indicates hypercompetition, in which the marked stem cell population expanded at the expense of the unmarked population, and a negative bias (down to -100%) indicates the converse. In cases of non-zero bias, the data can also reveal whether a bias might result from a change in the loss rate of marked FSCs, or from a change in invasion rate, or from both of these (Eq. (*) in Methods). In particular, hypercompetition (*b*>0) resulting from reduced stem cell loss will be characterized by less frequent stem cell replacement overall; while hypercompetition from increased stem cell invasion can give the same bias but will instead speed up stem cell replacement. Similarly, hypocompetition (*b*<0) can occur with increased or decreased stem cell replacement corresponding, respectively, to an increase in stem cell loss or a decrease in stem cell invasion. For a rigorous estimate of the model parameters, we evaluated their *Maximum Likelihood Estimators (MLE)*, and their 95% confidence intervals (95%CI) (see “Theory Supplement,” Text S1 and [24]) for both the loss/replacement rates and the bias. To make the approach widely applicable in other tissues, we also extended the biased competition model to niches with more than two stem cells (see Methods, “Theory Supplement,” Text S1).

As expected from our initial analysis, we found that our experimental data agreed extremely well with a model of wildtype FSCs undergoing neutral competition (Fig 1D and Table S3, *p*-value = 0.19 for the null hypothesis that there is zero bias, calculated by the Likelihood Ratio Test), and that wildtype FSCs have an overall average replacement time of 0.15/week (Table S2 and Fig S5). These observations confirm that wildtype FSCs undergo neutral competition for niche occupancy with loss and replacement of FSCs normally occurring in a stochastic manner.

### FSCs have a distinct cell polarity

To identify genes that regulate FSC competition, we performed a screen through the “Bruinfly” collection, which is a set of lines with molecularly defined lethal mutations that have been recombined onto the appropriate FRT for clone generation [25]. We used these lines to generate GFP^-^ homozygous mutant FSC clones in adult flies, and assayed for changes in clone frequency at two time points after clone induction. We found that the frequency of FSC clones produced by two lines, 111725 and 111512, dramatically increased between the two time points (data not shown). The P-elements in these lines are inserted near CG11377 and gliotactin, respectively. However, we also noticed that the mutant follicle cells produced by both of these lines had a defective cell polarity. Moreover, in a cross between the two lines, 111725/111512 transheterozygous progeny died as larvae. This was suspicious because an estimated 20% of the chromosome 2L Bruinfly stocks have second-site mutations in *lethal giant larvae* (*lgl*) [26], which is essential for the maintenance of cell polarity. Thus, we performed a complementation test and found that, indeed, 111725 and 111512 failed to complement a canonical *lgl* allele. This let us to the discovery that *lgl* regulates FSC competition for niche occupancy, as described below.

To explore whether *lgl* could be relevant to FSC competition, we assayed for the expression and localization of Lgl, as well as other cell polarity and cell adhesion proteins in FSCs and their early daughter cells. We used multiple methods to accurately identify FSCs. First, we induced LacZ^+^ mitotic clones in adult flies, allowed the clones to grow for at least 5 days, and restricted our analysis to ovarioles with mature FSC clones, defined as those that originate at the region 2a/2b border and include roughly half of the follicle cells in the germarium. In these ovarioles, FSCs can be reliably identified as the anterior-most labeled cell in the clone that is located on the side of the germarium [6]. Second, as a complementary approach, we identified FSCs using Notum-LacZ, a highly specific marker that we found to be upregulated in 65% of FSCs (and always absent from FSC daughter cells) [27]. Lastly, we used other features, such as low levels of FasIII expression and a triangular-shaped nucleus to further aid in the identification of FSCs.

**Figure 2:**
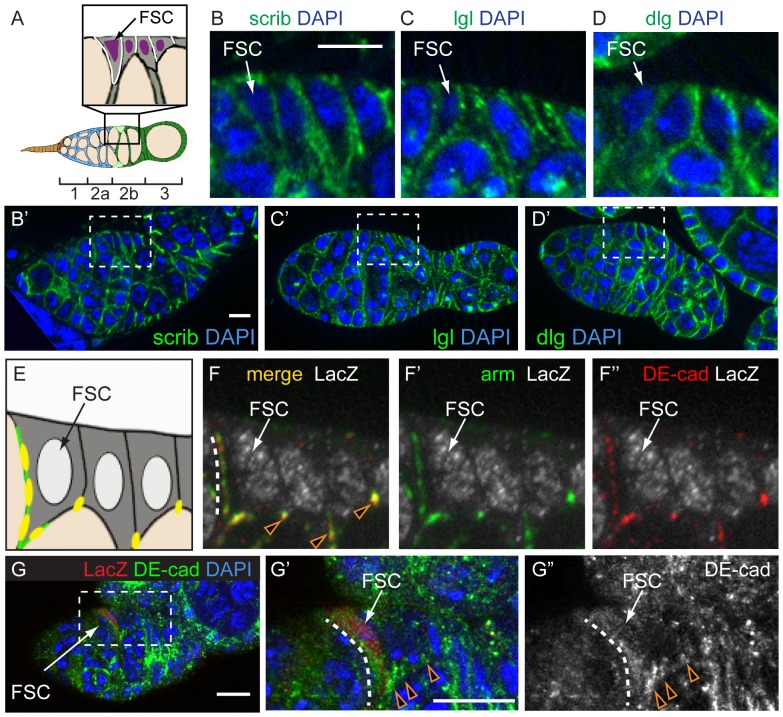
FSCs have basolateral junction proteins and broadly distributed adherens junctions. **A**. Diagram of the Drosophila germarium. Inset shows the localization of Lgl, Dlg and Scrib in follicle cells (white), which is similar in FSCs and downstream follicle cells. **B–D**. Wildtype germaria stained for Scrib (B), Lgl (C), or Dlg (D) (green) and DAPI (blue). The regions inside the dashed boxes in B′–D′ are magnified in B–D. **E**. Diagram summarizing the co-localization (yellow) of Arm (green) and DE-Cad in follicle cells. **F–G**. Germaria with a mature LacZ^+^ FSC clone (white) stained for Arm (green) and DE-Cad (red). **F**. Adherens junctions are distributed broadly in FSCs (white dotted line) and are restricted to the apical-lateral junctions in downstream follicle cells (orange triangles). The arm and DE-cad channels are shown separately with the LacZ channel in F′ and F″, respectively. **G**. A Notum-LacZ germarium stained for LacZ (red) to identify the FSC, DE-cad (green) and DAPI (blue). The boxed region in G is magnified in G′ and G″. Again, DE-cad is distributed in a broad streak along the anterior lateral surface of the FSC (white dotted line) and restricted to the apical-lateral junctions in downstream follicle cells (orange triangles). Anterior is to the left. Scale bar represents 5 µm.

While many features of cell polarity were similar in FSCs and their immediate daughter cells, called prefollicle cells, other features were distinct. For example, the basolateral junction proteins, Lgl, Dlg, and Scribble (Scrib), are present and localized specifically to the lateral surfaces in somatic cells throughout regions 2 and 3 of the germarium, which include the FSCs and prefollicle cells (Fig 2A–D). Likewise, we found that αPKC, Par-1, and moesin were expressed and had a similar pattern of diffuse localization around the cell membrane in both FSCs and prefollicle cells (Fig S1A–C). In addition, Gαi and patJ were undetectable in both FSCs and prefollicle cells, but became detectable in more mature follicle cells (Fig S1D–E), whereas partner of numb, brain tumor, and prospero appeared to be diffuse in or completely absent from FSCs and all follicle cells in the germarium (Fig S1F–H).

**Figure 3:**
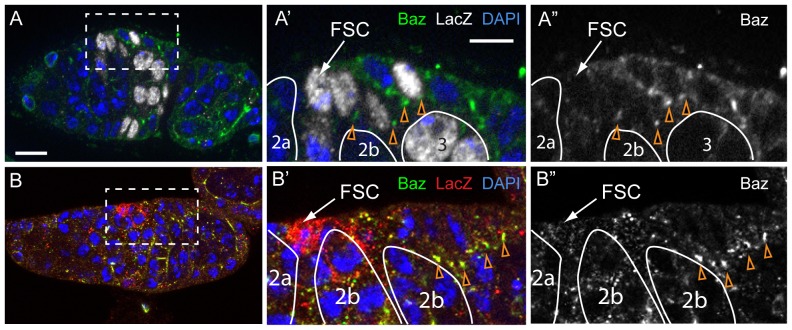
Bazooka is not localized to the apical domain of FSCs. **A**. A germarium with a mature LacZ^+^ FSC clone (white) stained for Baz (green) and DAPI (blue). Baz is distributed diffusely in the FSC and localized to the apical domain in downstream follicle cells (orange triangles). **B**. A Notum-LacZ germarium stained for LacZ (red) to identify the FSC, baz (green) and DAPI (blue). Again, baz is diffuse and difficult to detect in the FSC, but upregulated in the downstream follicle cells (orange triangles). The boxed regions in A and B are magnified in A′ and A″, and B′ and B″ respectively. A″ and B″ show the Baz channel only. Germ cell cysts are outlined in solid white lines and stages are indicated. Anterior is to the left. Scale bar represents 5 µm.

However, we also noticed a distinct difference in cell polarity between FSCs and prefollicle cells. Specifically, the two core components of adherens junctions, DE-Cad and Arm, were more broadly distributed in FSCs than in prefollicle cells (Fig 2E–G). DE-Cad and Arm double-positive puncta coated the entire anterior-lateral surface of FSCs (95% of FSCs, n = 20) but were restricted to the apical-lateral junction in prefollicle cells. High-resolution confocal microscopy of the FSC niche region in germaria with DE-cad::GFP verified that these DE-cad puncta are between FSCs and escort cells (Fig S2). In addition, we found that Baz is localized diffusely in FSCs (80.9% of FSCs, n = 21) but localizes to the presumptive apical surface of prefollicle cells (Fig 3). These observations indicate that the cell polarity of FSCs is partially distinct from their immediate daughter cells.

### *lgl* and *dlg* mutant FSCs are hypercompetitive for niche occupancy

**Figure 4:**
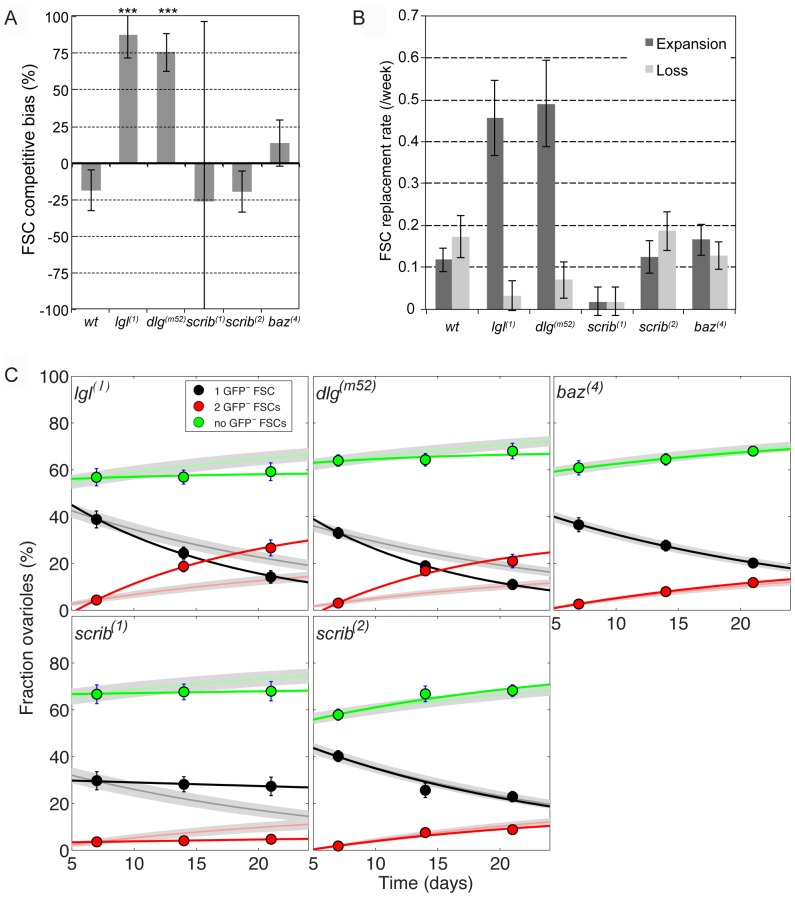
*lgl* and *dlg* mutant FSCs, but not *scrib* or *baz* mutant FSCs, are hypercompetitive. **A**. The competition bias of marked (wildtype or mutant) FSC clones for each genotype relative to the unmarked (wildtype) FSCs, inferred by fitting the clone dynamics to the FSC competition model (Fig. 1C and Methods). A bias of 0 indicates that the marked FSCs undergo neutral competition with the unmarked FSCs; a positive bias indicates that the marked FSCs are more competitive than the unmarked FSCs; and a negative bias indicates that the marked FSCs are less competitive than the unmarked FSCs. Error bars = S.E. Stars indicate that the bias is significantly different from zero (*p*<0.001, Table S3). **B**. The rate of replacement of marked (wildtype or mutant) FSCs and unmarked (wildtype) FSCs for each genotype is similarly obtained from the model. The expansion/loss rates correspond respectively to *r*_+_, *r*_-_ in Fig. 1C. Error bars = S.E. **C**. The observed and predicted fraction of ovarioles with 0, 1 or 2 GFP^-^ FSCs at 7, 14 and 21 dphs for each genotype. Data points indicate experimental measurements (error bars = S.E.), faint lines and grey regions indicate the mean and 95% confidence intervals assuming no change in FSC behavior from wildtype, except for labeling efficiency. Solid lines show the Maximum Likelihood Fits with changes in both the competitive bias and the average FSC replacement rate according to the parameter values in panels A,B. The observed values for *lgl^1^* and *dlg^m52^* deviate substantially from the predicted values whereas the observed values for *scrib^2^* and *baz^1^* are closely aligned with the predicted values. The observed values for *scrib^1^* deviate substantially from the predicted values because the replacement rate, but not the bias, is significantly different from wildtype.

We next investigated whether loss of specific basolateral junction proteins influenced the rate of FSC replacement. In ovarioles with clones that were mutant for *lgl^1^*, a strong loss-of-function allele, we found that the frequency of GFP^-^ (*lgl^1/1^*) FSCs increased 1.4 fold (Table 1). We quantified the frequency of ovarioles with 0, 1, or 2 *lgl^1/1^* FSCs at each time point and analyzed the data using the competition model described above. We found that the frequency of mosaic ovarioles decreased from 7 to 21 dphs, as expected for FSC competition, yet nearly all (92%) of this decrease was accounted for by an increase in the frequency of uniformly marked (GFP^-^, *lgl^1/1^*) ovarioles. These values deviate significantly from those expected if competition were neutral (Fig. 4C and Table S3, *p*-value<10^-6^). Indeed, we find that *lgl^1/1^* FSCs had a strong positive competitive bias (90%, Fig. 4A) over the three time points tested (95%CI = [63%,99%]). This positive bias arose through both a drop in the rate of mutant FSC loss and an increase in the rate at which mutant FSCs displaced their wildtype neighbors (Fig 4B and Table S1). Together, these changes led to an overall faster rate of FSC replacement in ovarioles with *lgl^1/1^* FSCs (0.25/week, versus 0.15/week in wildtype, Table S2).

In ovarioles with clones that were mutant for *dlg^m52^*, a strong hypomorph that behaves as a null allele, the frequency of GFP^-^ (*dlg^m52/m52^*) FSCs also increased 1.4 fold from 7 dphs to 21 dphs (Table 1), again indicating biased competition of the mutant FSCs. As with *lgl^1/1^* mutants, the frequency of mosaic ovarioles decreased over the same period and most of this decrease (82%) was accounted for by the increase in the frequency of uniformly marked (GFP^-^, *dlg^m52/m52^*) ovarioles. Again, these values deviate significantly from neutral competition (Fig. 4C, Table S3, *p*-value<10^−7^), and our analysis indicates that *dlg^m52/m52^* FSCs had a strong positive competitive bias (79%, Fig. 4A) over the three time points tested (95%CI = [53%,93%]). As with *lgl^1/1^* mutants, this positive bias arose through a significant drop in the mutant FSC loss rate and a large increase in the rate at which wildtype FSCs are lost (Fig. 4B and Table S1), and it resulted in an overall faster FSC replacement rate (0.28/week, versus 0.15/week in wildtype, Table S2). Therefore, *dlg^m52/m52^* FSCs are also strongly hypercompetitive for niche occupancy.

### Loss of Scrib or Baz did not cause hypercompetition

In ovarioles with clones that were homozygous for loss-of-function alleles of *scrib*, the frequency of GFP^-^ FSCs remained approximately constant (18% for *scrib^1^* and 20–22% for *scrib^2^*, between 7 and 21 dphs, Table 1). In addition, the frequencies of uniformly marked and unmarked ovarioles increased by roughly equal amounts from 7 to 21 dphs over this same time period. The data for *scrib^2^* conform to the neutral drift model (Fig. 4A,C, Table S3, *p*-value = 0.18), and we also found no significant competition bias for *scrib^1^* (*p*-value>0.999), though the overall rate of FSC replacement in ovarioles with *scrib^1/1^* clones was extremely low (Fig. 4B, Table S2), so we would need to assay substantially more ovarioles to obtain an accurate estimate of the competition bias for this allele.

Next, because Baz localization is one of the earliest detectable differences in cell polarity between FSCs and their daughters (Fig. 3), we considered whether *baz* mutant FSCs were hypercompetitive. In ovarioles with *baz^4^* FSC clones, the frequency of GFP^-^ (*baz^4/4^*) FSCs remained constant (21–22%) at 7, 14 and 21 dphs (Table 1). In addition, the frequencies of uniformly marked and unmarked ovarioles increased by similar amounts from 7 to 21 dphs (9% and 8%, respectively, Table 1) over this same time period. These data are consistent with neutral competition (Fig. 4A,C, Table S3, *p*-value = 0.42), although it is possible that *baz^4/4^* FSCs are slightly hypercompetitive (bias = 13±16%, MLE±SE). Lastly, the presence of the *baz^4/4^* clones in the population had little effect on the overall rate of FSC replacement (Fig. 4B, Table S2). Collectively, this suggests that the roles of Lgl and Dlg in FSC competition are not dependent on the role of Baz in the establishment of apical cell polarity, and consequently *baz^4/4^* FSCs undergo neutral competition with wildtype FSCs.

### Hypercompetitive bias is not associated with increased proliferation rate

Lgl, Dlg, and Scrib are tumor suppressors that prevent follicle cell neoplasia in mid-oogenesis [28]. Therefore, to determine whether an increase in the rate of proliferation was associated with hypercompetition in the FSC niche, we assayed for changes in tissue architecture or proliferation in ovarioles with *lgl*, *dlg*, or *scrib* mutant FSC clones. We observed follicle cell neoplasia in *lgl^1^*, *dlg^m52^*, and *scrib^1^* clones in mid-oogenesis, as has been reported previously [28], [29], but mutant follicle cells in germaria of the same ovarioles generally had a normal tissue architecture (Fig S3).

To compare the rates of mitosis between wildtype and mutant follicle cells, we determined the frequencies of phosphohistone H3^+^ follicle cells in germaria from ovaries with mature wildtype, *lgl^1^*, *dlg^m52^*, or *scrib^2^* FSC clones. Although we could not distinguish between FSCs and prefollicle cells in these analyses, the FSCs and prefollicle cells have similar rates of division (approximately 12 and 9.6 hours per cell cycle respectively), and prefollicle cells represent the majority of cells in the germarium from the FSC lineage. Thus, these measurements are likely to be a good approximation of prefollicle cell division rates. In each case, we found that the frequency of phosphohistone H3^+^ cells was comparable between the heterozygous (GFP^+^) and homozygous mutant (GFP^-^) follicle cell populations (Table 2). Therefore, the competitive advantage of *lgl^-^* and *dlg^-^* prefollicle cells is not likely to be due to an increased proliferation rate.

**Table 2:** Quantification of phosphohistone H3^+^ follicle cells in the germarium.

	GFP^-^ (wildtype or mutant)	GFP^+^ (wildtype)	Fisher Exact (p-value)
Wildtype	15% (41)	14% (71)	0.79
lgl(1)	8% (51)	14% (95)	0.42
dlg(m52)	11% (27)	15% (227)	0.78
scrib(2)	15% (55)	14% (153)	>0.999

Values indicate the percent of GFP^-^ or GFP^+^ follicle cells that are phosphohistone H3^+^ in germaria with GFP^-^ clones of the indicated genotypes. The GFP^-^ cells are either wildtype (first row) or heterozygous for the genotype indicated. The total number of observations in each case are in parentheses. A Fisher Exact Test value of p>0.05 indicates that there is no significant association between labeling status (GFP^-^ or GFP^+^) and the presence of a phosphohistone H3 signal.

### Hypercompetitive follicle cells accumulate in the FSC niche region

**Figure 5:**
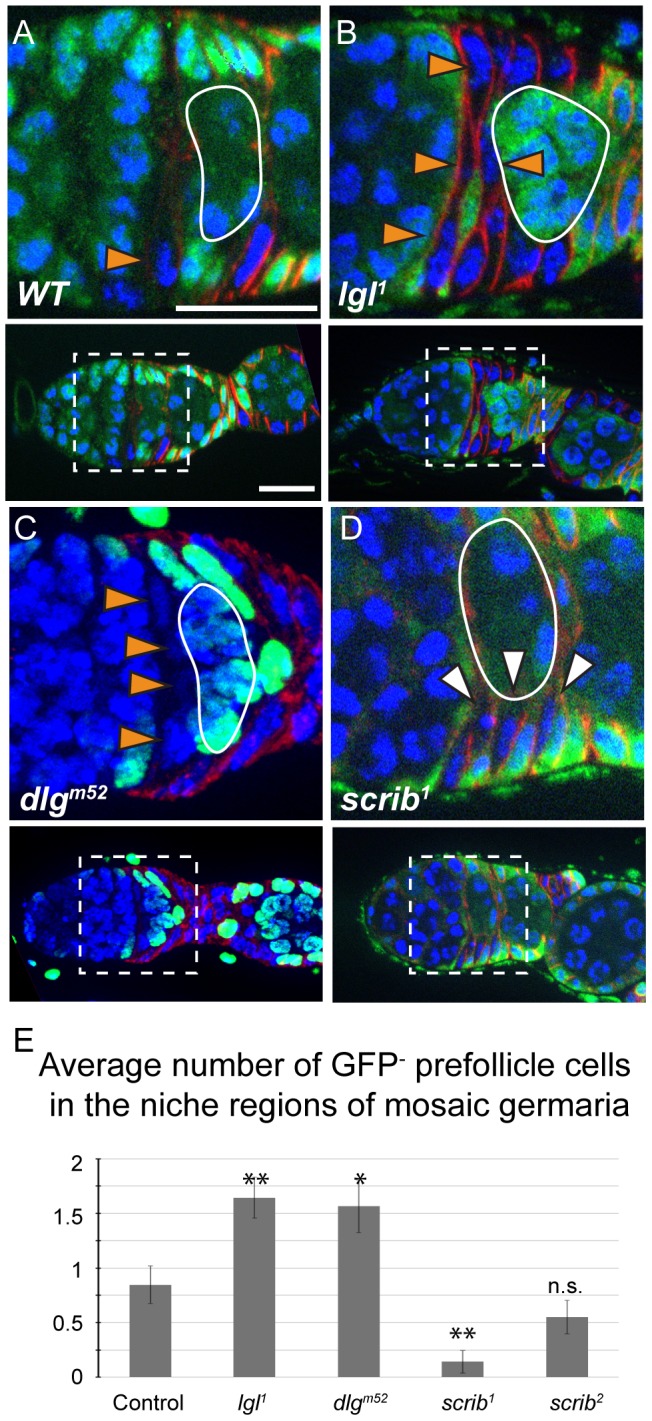
Hypercompetitive prefollicle cells accumulate in the FSC niche region. **A–D**. Germaria with mature GFP^-^ wildtype (A), *lgl^1/1^* (B), *dlg^m52/m52^* (C), or *scrib^1/1^* (D) FSC clones 14 days post heat shock stained for FasIII (red), GFP (green), and DAPI (blue). Wildtype clones typically contained only one GFP^-^ prefollicle cell along the Region 2a/2b border (orange triangle, A) whereas *lgl^1/1^* and *dlg^m52/m52^* clones often contained 2 or more GFP^-^ prefollicle cells in this region (orange triangles, B and C). *scrib^1/1^* clones typically produced prefollicle cells that only migrated along the side of the germarium (white arrows) and rarely produced prefollicle cells that migrated along the Region 2a/2b border. Images of the full germarium is shown in the panel below each magnified region. Anterior is to the left. Scale bar represents 5 µm. **E**. Quantification of the average number of GFP^-^ (wildtype or mutant) prefollicle cells along the Region 2a/2b border in mosaic germaria of the indicated genotypes. Error bars represent S.E. and significance was determined using a two-tailed Students T-test. * indicates p<0.05 and ** indicates p<0.01.

Approximately half of the FSC divisions produce a daughter cell that migrates along the Region 2a/2b border and contacts the FSC on the opposite side of the germarium [17]. These daughter cells then either enter the niche as part of an FSC replacement event or, more frequently, move away from the FSC niche region and begin to differentiate. Biased competition of *lgl* and *dlg* mutants could therefore result from a defect that increased the number of opportunities that mutant progeny have to invade the niche, or from a defect that increased the advantage of mutant progeny compared to wildtype at each opportunity. To investigate these possibilities, we quantified the number of GFP^-^ prefollicle cells along the Region 2a/2b border in mosaic germaria (i.e. germaria with one GFP^-^ FSC that is either wildtype or homozygous mutant for a gene of interest, and one GFP^+^ FSC). We found significantly more GFP^-^ prefollicle cells migrating along the Region 2a/2b border in germaria with *lgl^1/1^* or *dlg^m52/m52^* clones compared to germaria with wildtype clones (Fig. 5A–C, E). In contrast, the average number of GFP^-^ prefollicle cells along the Region 2a/2b border in germaria with *scrib^2^* follicle cells was slightly lower, but not significantly different than wildtype (Fig. 5E).

Interestingly, we found that *scrib^1/1^* FSCs rarely produced cross-migrating prefollicle cells (Fig. 5D–E), which may explain why we observed so little replacement of the GFP^+^ FSCs in these germaria. Similarly, the presence of a single *scrib^1^* allele in the GFP^+^ prefollicle cells, or perhaps a non-autonomous effect of *scrib^1/1^* clones in the tissue, may have reduced the frequency of cross-migrating GFP^+^ cells in these germaria, which would explain the reduction in the rate of replacement of GFP^-^ FSCs. However, we cannot accurately track the cross-migrations of the GFP^+^ prefollicle cells because they are difficult to distinguish from nearby GFP^+^ escort cells. Therefore, the number of prefollicle cells in migrating along the Region 2a/2b border in germaria with *lgl*, *dlg* or *scrib* mutant clones correlates with the rate of FSC replacement.

### Hypercompetitive follicle cells are delayed in differentiation

**Figure 6:**
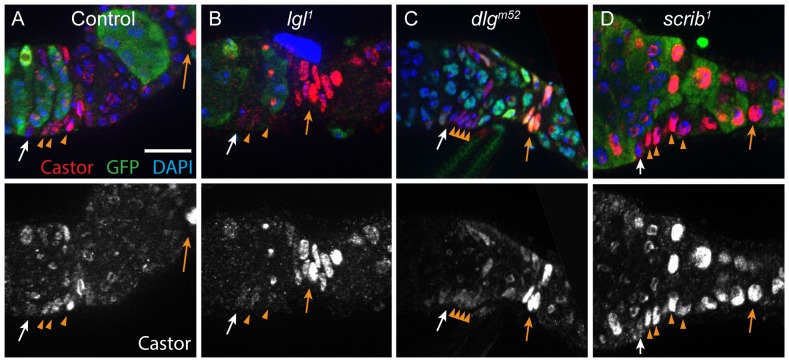
Hypercompetitive prefollicle cells have reduced *castor* expression. Germaria with mature wildtype (A), *lgl^1^* (B), *dlg^m52^* (C), or *scrib^1^* (D) FSC clones stained for castor (red), GFP (green) and DAPI (blue). In wildtype and *scrib^1^* clones, *cas* expression is low in FSCs (white arrows) and higher in prefollicle cells (orange arrowheads) whereas in *lgl^1^* and *dlg^m52^* clones, *cas* expression is low in both FSCs (white arrows) and prefollicle cells (orange arrowheads). The uniformly bright polar and stalk cells (orange arrows) within clones of all genotypes served as a control for antibody staining and exposure times across samples. In addition, the high levels of *cas* expression in *lgl^1^* and *dlg^m52^* polar and stalk cells, indicate that *cas* expression, though delayed, eventually reaches normal levels in these mutant clones.

Our observation that *lgl^-^* and *dlg^-^* prefollicle cells accumulate in the FSC niche region suggested that these mutant cells are delayed in differentiation. To further test this possibility, we examined the expression of *castor* (*cas*), a transcription factor that is required for proper differentiation of the early FSC lineage [30], in wildtype and mutant FSC clones. Consistent with published studies [30], we found that, in wildtype germaria, *cas* is expressed at low levels in FSCs, at higher levels in prefollicle cells, and then becomes restricted to the polar/stalk lineage in more mature follicles (Fig. 6A). In contrast, we found that *cas* expression remained low in *lgl^1^* and *dlg^m52^* prefollicle cells (Fig. 6B–C), whereas *scrib^1^* prefollicle cells exhibited a wildtype pattern of *cas* expression (Fig. 6D). Likewise, we found that the expression of *cas* was lower in the prefollicle cells of germaria in which *lgl* or *dlg* RNAi was expressed under the control of a follicle cell driver (109-30-Gal4) than in germaria in which *scrib* RNAi was expressed under the control of the same driver (Fig. S4). Collectively, these results suggest that *lgl^-^* and *dlg^-^* clones are hypercompetitive because they have a differentiation defect that permits them to migrate to the opposite niche but delays their entry into the follicle epithelium, which would carry them away from the niche.

## Discussion

Here, we have demonstrated that FSCs normally undergo neutral competition through stochastic replacement events, and that *lgl^-^* and *dlg^-^* FSCs undergo non-neutral hypercompetition with wildtype FSCs. Our analysis allowed us to probe several aspects of this interaction. First, we developed a metric for competition bias, which measures the frequency that stem cells in a marked population undergo symmetric renewal versus differentiation. Since the total number of stem cells in the tissue remains constant, the expansion of a marked subset of stem cells (due to an increase in the frequency of symmetric renewal) causes an equal contraction of the unlabeled stem cell pool. Thus, the competition bias is also a measure of the effects that influence the relative abundance of the marked and unmarked stem cells within the tissue.

Our quantitative method is similar to the method used in two recent studies of stem cell replacement in the mouse intestine [31], [32], but extends to a more general case of stem cell competition (“Theory Supplement,” Text S1), including but not requiring nearest-neighbor stem cell interactions. In addition, our method provides a framework for Bayesian parameter estimation from just three clonal states (mosaic, fixed and extinct), which allow the competition parameters to be inferred from experimentally accessible long-term clonal dynamics. Moreover, our method provides independent measurements of competition bias and competition rate, which allowed us to distinguish between mutations with different effects. For example, we found that *scrib^1^* mutations strongly decreased competition rate whereas *lgl^1^* and *dlg^m52^* mutations increased both competition rate and competition bias. Lastly, we developed formal hypothesis tests for the changes in these rate/bias metrics that allowed us to separate insignificant changes (such as those caused *baz^4^*) from significant changes.

Our measurements of the rate of stem cell replacement indicate the frequency that stem cells in the tissue undergo symmetric versus asymmetric divisions. In all cases, just a few percent of FSC divisions are symmetric. The factors that govern the rate of replacement are not fully understood, but limiting niche space is likely to play a role in epithelial tissues. For example, if a stochastic event causes a stem cell to differentiate and vacate the niche, this would require the daughters of a neighboring stem cell to acquire symmetric fates to fill the empty niche space. Interestingly, our observation that the rate of replacement increases in ovarioles with hypercompetitive FSCs implies that the converse is also true. That is, hypercompetitive FSCs undergoing symmetric renewal can increase the chance that the neighboring wildtype FSCs will differentiate and vacate the niche.

Our observations about FSC polarity reveal at least two new aspects of the process by which FSC and daughter cell fates diverge. First, our observation that the immediate FSC daughter cells have a different pattern of cell polarity than FSCs indicates that the stepwise process of apical-basal polarization [33] begins as soon as the daughter cells are formed, and that this process is important for resolving competition for the FSC niche. It will be interesting to explore whether the differences in cell polarity are a cause or a consequence of the divergence in FSC and daughter cell fates. Second, since FSC daughter cells are still capable of re-entering the niche [17], these early steps must be reversible in the case of an FSC replacement event.

Lgl, Dlg, and Scrib form a complex that regulates apical-basal polarity and have similar loss-of-function phenotypes in embryonic and larval epithelia [28]. Thus, it was surprising to find that *lgl^-^* and *dlg^-^* FSCs behaved differently from *scrib^-^* FSCs. Interestingly, however, *lgl^-^* and *dlg^-^* follicle cells are more invasive [34] and migrate more rapidly [35] than *scrib^-^* follicle cells. These studies suggest that Lgl and Dlg are part of a Scrib-independent complex that prevents follicle cells from disassociating from the follicle epithelium and invading between germ cells [35]. Like these invading follicle cells, prefollicle cells on the Region 2a/2b border are also migratory and not fully associated with the follicle epithelium. Thus, a defect in the ability to associate with the follicle epithelium may also explain why *lgl^-^* and *dlg^-^* prefollicle cells are delayed in differentiation (Fig. 6) and accumulate along the Region 2a/2b border (Fig. 5). This process does not seem to depend on Scrib function since FSCs that were homozygous mutant for *scrib^2^*, a molecular null, produced clone patterns that were similar to wildtype. *scrib^1^* is also a strong loss-of-function allele, but it may retain function in some circumstances [36]. Thus, the large reduction in the frequency of cross-migrating prefollicle cells in *scrib^1^* FSC clones (Fig. 5) may be a neomorphic phenotype caused by this allele.

Collectively, these observations suggest that Lgl and Dlg function in a Scrib-independent manner to suppress hypercompetition by promoting the incorporation of prefollicle cells into the growing follicle epithelium. Because prefollicle cells that lack *lgl* or *dlg* accumulate in the FSC niche region, these cells may have more opportunities to re-enter the niches on either side of the germarium, which could be the cause of both the increase in the rates of mutant clone expansion and the decrease in the rates of mutant clone loss that we observed. Specifically, an increase in the rate of entry into the niche containing the wildtype FSC would cause an increase in the rates of mutant clone expansion whereas an increase in the rate of re-entry into the niche that the mutant prefollicle cell originated from may decrease the chance that this niche is occupied by a wildtype prefollicle cell. Further studies of niche competition will continue to provide insight into the mechanism by which a healthy population of stem cells are maintained and how this process fails in the prelude to chronic diseases such as cancer.

## Methods

### Drosophila stocks and clonal analysis

Drosophila stocks were maintained at 20–25°C. The following genotypes were used to generate clones:

#### β-gal+ positive clones

*yw, hsFlp; X.15.29/X.15.33* [37].

#### GFP- clones

(1) *hsFlp/+; FRT 40a/Ubi-GFP, FRT 40a*, (2) *hsFlp/+; lgl^1^, FRT 40a/Ubi-GFP, FRT 40a,* (3) *hsFlp/+; lgl^4^, FRT 40a/Ubi-GFP, FRT 40a,* (4) *dlg^m52^, FRT 101/histone-GFP, FRT 101; ; MKRS(hsFlp)/+,* (5) *hsFlp/+; ; scrib^1^, FRT 2a, FRT 82b/Ubi-GFP, FRT 82b, *(6) *hsFlp/+; ; e, scrib^2^, FRT 82b/Ubi-GFP, FRT 82b, *(7) *FRT 9-2/Ubi-GFP, FRT 9-2; Pr, Pwn, hsflp/+and* (8) *baz^4^, FRT 9-2/Ubi-GFP, FRT 9-2; Pr, Pwn, hsflp/CyO.*

#### RNAi

(1) *scrib*: y^1^ v^1^; P{y^+t7.7^ v^+t1.8^, TRiP.JF03229}attP2, (2) *lgl*: y^1^ v^1^; P{y^+t7.7^ v^+t1.8^, TRiP.JF01553}attP2, and (3) *dlg*: y^1^ v^1^; P{y^+t7.7^ v^+t1.8^, TRiP.JF01365}attP2.

Oregon-R-C was used as wildtype. Baz-GFP is CC01941 in the Carnegie Protein Trap Collection [38]. DE-Cad::GFP is described in [39]. The FSC LacZ reporter is yw; Notum-LacZ, described in [40]. 109-30-Gal4 is described in [41]. All stocks were obtained from the Bloomington Stock Center except CC01941, which was obtained from Allan Spradling; *yw; lgl^1^, FRT 40a*, *w; e, scrib^2^, FRT 82B/TM6c and scrib^3^ FRT 2a, FRT82b/TM3, ftz, LacZ*, which were obtained from David Bilder; and *yw, baz^4^, FRT 9-2/FM6, ywB; SPT/CyO*, *yw, Ubi-GFP, FRT 9-2/FM6; Pr, Pwn hsflp/Cyo* and *FRT 9-2*, which were obtained from Yuh Nung Jan. Further information on symbols and genes can be found in Flybase (http://flybase.bio.indiana.edu) and the Bloomington *Drosophila* Stock Center (http://fly.bio.indiana.edu/).

Mitotic clones were generated by placing adult flies in bottles with wet yeast for at least 2 days, heat shocking flies twice a day for two days (4 times total) for 1 hour in a 37°C water bath, and then maintaining flies on wet yeast, changed daily, at 25°C for up to 21 dphs.

### Immunostaining and fluorescence microscopy

Ovaries were dissected and stained as described previously [17]. Briefly, ovaries were dissected in Graces medium (Gemimi Bio-Products), fixed in 4% paraformaldehyde (Sigma), diluted from a 20% solution in 1x PBS for 10 minutes, and blocked with 1x PBST (PBS+0.2% Triton X-100). Fixed tissues were incubated with primary antibody overnight at 4°C, rinsed and washed for 1 hr, incubated with secondary antibody at room temperature for 2 hours, rinsed and washed for 1 hr, stained with DAPI for 5 minutes, rinsed with 1x PBS and mounted on microscope slides with Vectashield (Fisher). 1x PBST was used for all washes and antibody dilutions. For immunostaining with anti-DE-cad antibody, the protocol was modified as follows. Ovaries were fixed in 5.3% paraformaldehyde, diluted from a 16% stock (Electron Microscopy Sciences) and the PBST used for washes was PBS+0.3% Triton X-100. FSCs were identified as the anterior-most labeled cell that is located on the side of the germarium in a mature clone. Low levels of FasIII expression and a triangular-shaped nucleus were used to further aid in the identification of FSCs. CMCs were identified as labeled cells in a mature clone that are at the region 2a/2b border in the middle of the germarium.

The following primary antibodies were used: guinea pig anti-Scrib 1∶500 (a gift from David Bilder), rabbit anti-Lgl 1∶250 (a gift from Juergen Knoblich), rabbit anti-Bazooka 1∶1000 (a gift from Andreas Wodarz), rabbit anti-Patj 1∶1000 (a gift from Hugo Bellen), rabbit anti-moesin 1∶1000 (a gift from Dan Kiehart), rabbit anti-Gαi 1∶200 (a gift from Juergen Knoblich), rabbit partner of numb 1∶1000 (a gift from Yuh Nung Jan), rabbit anti-brat 1∶1000 (a gift from Nahum Sonenberg) guinea pig anti-traffic jam (a gift from Allan Spradling) and rabbit anti-castor 1∶5000 (a gift from Ward Odenwald). Also, we used mouse anti-Dlg 1∶50, mouse anti-Armadillo 1∶4, rat anti-DE-Cadherin 1∶100, mouse anti-Hu li tai shao 1∶50, and mouse anti-FasIII 1∶20 from the Developmental Studies Hybridoma Bank; rabbit anti-GFP 1∶5000 (Torrey Pines Biolabs); mouse anti-GFP 1∶1000 (Invitrogen); rabbit anti-αPKC 1∶500 (Santa Cruz Biotechnology); mouse anti-phosphohistone H3 1∶1000 (Cell Signaling) and rabbit anti-cleaved caspase 3 1∶200 (Cell Signaling). Goat anti-rabbit and goat anti-mouse secondary antibodies conjugated to Alexafluor 488, 555, or 633 (Invitrogen) were used at 1∶1000. DAPI (Sigma) was prepared in 1x PBS at 1 µg/ml. Phalloidin (Invitrogen) was used at 1∶200 and was added to the secondary antibody solution.

All images were acquired on a Zeiss M2 Axioimager with apotome or Nikon Eclipse Ti spinning disc confocal. Images were processed using Adobe Photoshop and figures were prepared in Adobe Illustrator. Adjustments to brightness, contrast, and gamma were applied to every pixel in the image.

### Stem cell competition model and statistical analysis

To analyze the fate outcomes of the marked FSCs, we assume that FSC replacement events occur relatively infrequently and in a stochastic manner. Denoting a labeled FSC as *L* and an unlabeled FSC as *U*, the two possible outcomes of FSC replacement can be characterized by the rates of invasion 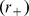
and loss 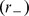
of the labeled stem cell:
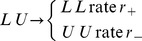
It has been argued that each ovary contains just two FSCs. If only one FSC is labeled, then replacement of either FSC will lead all ovaries to eventually contain either two labeled FSCs (*LL*), or none (*UU*) (Margolis and Spradling 1995). This model is perhaps the simplest example of a Moran process (Ewens, 2004), with the same features as previous models of stem cell replacement (Klein et al., Cell Stem Cell, 2010; Klein and Simons, 2011), but greatly simplified by the presence of just two FSCs. Denoting *f_n_*(*t*) as the fraction of ovaries containing *n* = 0,1,2 labeled FSCs at a time *t*, the model dynamics are,
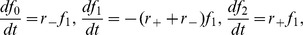
which have the solution,
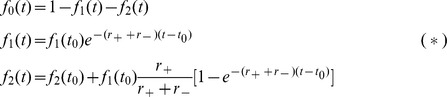
Here *t*_0_ corresponds to the first experimental time point. In the case of neutral competition, the rates of FSC invasion and loss are precisely balanced, i.e. *r_+_ = r_–_*. In cases where *r_+_* or *r_–_* are altered by the effect of mutation, we may distinguish between changes in the average replacement rate, *R* = (*r_+_+r_–_*)/2, and the competitive bias of the labeled FSC compared to its unlabeled neighbor, which we define as *b* = (*r_+_–r_–_*)/(*r_+_+r_–_*).

Although these results have been defined for just two FSCs, if more than two stem cells compete, the same quantitative behavior [Eq. (*)] emerges in the long-term clonal dynamics, but now *f*_0,1,2_(*t*) correspond respectively to the fraction of unlabeled, mosaic, and fully-labeled ovaries (“Theory Supplement,” Text S1). Therefore, the fit to the data and identification of a competitive bias is independent of the number of stem cells in the niche. For a stem cell count *N*_S_ that is greater than two, the actual bias is related to the effective value inferred by fitting to Eq. (*) through the relation,


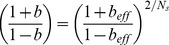
. The average replacement time is related to that inferred from Eq. (*) though the relation 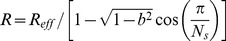 for the case where stem cells are replaced by their nearest neighbors; for the case where any stem cell in the niche may replace another, one finds 
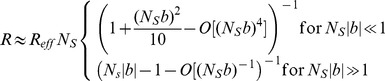
, (see “Theory Supplement,” Text S1). Bayesian methods for parameter estimation and null hypothesis testing are provided in the “Theory Supplement,” Text S1.

## Supporting Information

Figure S1 **Many polarity markers have a similar pattern of expression and localization in FSCs and downstream daughter cells.** **A**. Par-1-GFP stained for GFP (green), FasIII (red), and DAPI (blue). **B–G**. Wildtype germaria stained for FasIII (red), DAPI (blue) and αPKC (B), moesin (C), PatJ (D), Gαi (E), partner of numb (F), or brain tumor (G) (green). A′–G′ shows the green channel only. **H**. A wildtype germarium (H) and intestine (H′) stained for prospero (green) and DAPI (blue). We observed no staining in the germarium and so used the intestine as a positive control for staining. White arrowheads indicate prospero^+^ enteroendocrine cells. FSCs are marked with asterisks. White triangle in E indicates Gαi staining in a follicle cell. Anterior is to the left. Scale bar represents 10 µm.
(TIF)

Figure S2 **DE-cad is distributed broadly along the region of contact between FSCs and escort cells.** **A–C**. *DE-cad::GFP* germarium stained for GFP (green, panel A) to label DE-cad, and phalloidin (red, panel A) to label cell membranes. The GFP channel and phalloidin channels are shown separately in B and C, respectively. The niche region (boxed in A–C) is magnified in A′–C′. A broad streak of DE-cad (orange dotted line in A′–B′) is visible on the anterior surface of the anterior most follicle cell, which is likely to be an FSC, whereas DE-cad is restricted to small puncta in the apical-lateral region of more posterior follicle cells (orange triangles). At this resolution, the surface of the escort cell (EC) that contacts the 2a cyst can be distinguished from the surface that contacts the FSC, revealing that the streak of DE-cad is between an FSC and EC. **D**. Germaria with a mature LacZ^+^ clone stained for DE-cad (green) and LacZ (purple). The FSC is identified as the anterior most LacZ^+^ cell in the clone. A broad streak of DE-cad is present on the anterior surface of the cell (orange triangles), which can be distinguished from the surface of the escort cell that contacts the 2a cyst. The niche region (boxed in D) is magnified in D′. Images were acquired using a Nikon spinning disc confocal microscope with a CFI Apo TIRF 100x lens (N.A.: 1.49). Anterior is to the left. Scale bar represents 5 µm.
(TIF)

Figure S3 **Dlg, Lgl, and Scrib mutations cause polarity defects but not hyperproliferation in the FSC niche region.** **A–C**. Germaria with mature GFP^-^ *lgl^1^* (A), *dlg^m52/m52^* (B), or *scrib^1^* (C) FSC clones 14 days post heat shock stained for GFP (green), FasIII (red), and DAPI (blue). A′, B′ and C′ show follicles from the same ovarioles shown in A, B, and C, respectively. Thus, the follicle epithelium looks normal in the germarium, even at time points when significant neoplasia is observed in downstream follicles. Anterior is to the left. Scale bar represents 5 µm.
(TIF)

Figure S4 **Knockdown of** ***lgl*** **or** ***dlg*** **but not** ***scrib*** **causes a decrease of** ***cas*** **expression in prefollicle cells.** Germaria in which UAS-scrib^RNAi^ (A), UAS-lgl^RNAi^ (B), or UAS-dlg^RNAi^ (C) expression is driven by 109-30-Gal4, a follicle cell driver, stained for cas (red) and DAPI (blue). *cas* expression levels in prefollicle cells (white lines) are substantially reduced in germaria expressing UAS-lgl^RNAi^ or UAS-dlg^RNAi^ compared to prefollicle cells in germaria expressing UAS-scrib^RNAi^. The consistently high expression of *cas* in stalk cells (orange arrows) served as a control for antibody staining and exposure times across samples.
(TIF)

Figure S5 The Likelihood function. Parameter estimation for one data set (wild-type), showing the Likelihood function L(R,b), and the projected Likelihood for the two model parameters. The red lines indicate the maximum Likelihood estimates (MLE) of the parameters, and blue lines show the 95% confidence interval.
(TIF)

Table S1 **The maximum likelihood estimates of expansion rates (r_+_) and loss rates (r_-_) of mutant FSCs, normalized to wildtype. SE indicates the Standard Error.**
(DOCX)

Table S2 **The maximum likelihood estimates (MLE) of the overall FSC replacement rate per week in germaria with FSC clones of the indicated genotypes.** The standard errors and 95% confidence intervals are provided.
(DOCX)

Table S3 **The maximum likelihood estimates (MLE) for the competitive bias of marked FSCs.** The standard errors and 95% confidence intervals are provided and the *p*-values are for the null hypothesis that competition is neutral (zero bias).
(DOCX)

Text S1 **Supplemental information describing the mathematical theory and modeling.** Additional information provided about (1) the maximum likelihood parameter estimation; (2) null hypothesis testing; and (3) A generalized model for competitive bias in a multi-stem cell niche.
(PDF)
