# Supplementary figures and images for: Basolateral Junction Proteins Regulate Competition for the Follicle Stem Cell Niche in the Drosophila Ovary
Source: PLoS One. 2014 Jul 3;9(7):e101085. doi: 10.1371/journal.pone.0101085 (PMC4084627; doi:10.1371/journal.pone.0101085)

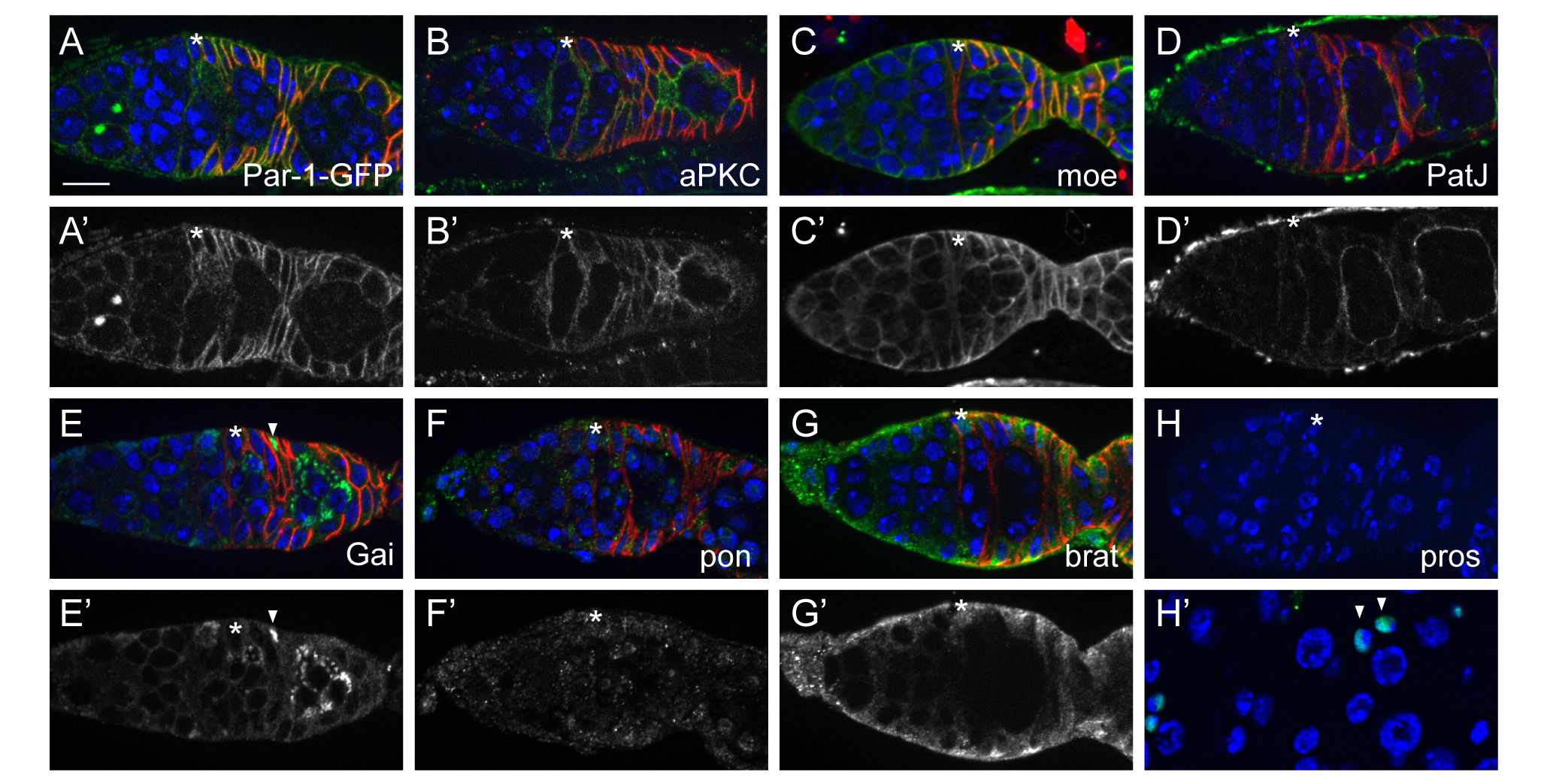

Supplement: Figure S1 — Many polarity markers have a similar pattern of expression and localization in FSCs and downstream daughter cells. A. Par-1-GFP stained for GFP (green), FasIII (red), and DAPI (blue). B–G. Wildtype germaria stained for FasIII (red), DAPI (blue) and αPKC (B), moesin (C), PatJ (D), Gαi (E), partner of numb (F), or brain tumor (G) (green). A′–G′ shows the green channel only. H. A wildtype germarium (H) and intestine (H′) stained for prospero (green) and DAPI (blue). We observed no staining in the germarium and so used the intestine as a positive control for staining. White arrowheads indicate prospero+ enteroendocrine cells. FSCs are marked with asterisks. White triangle in E indicates Gαi staining in a follicle cell. Anterior is to the left. Scale bar represents 10 µm. (TIF) [file pone.0101085.s001.tif]

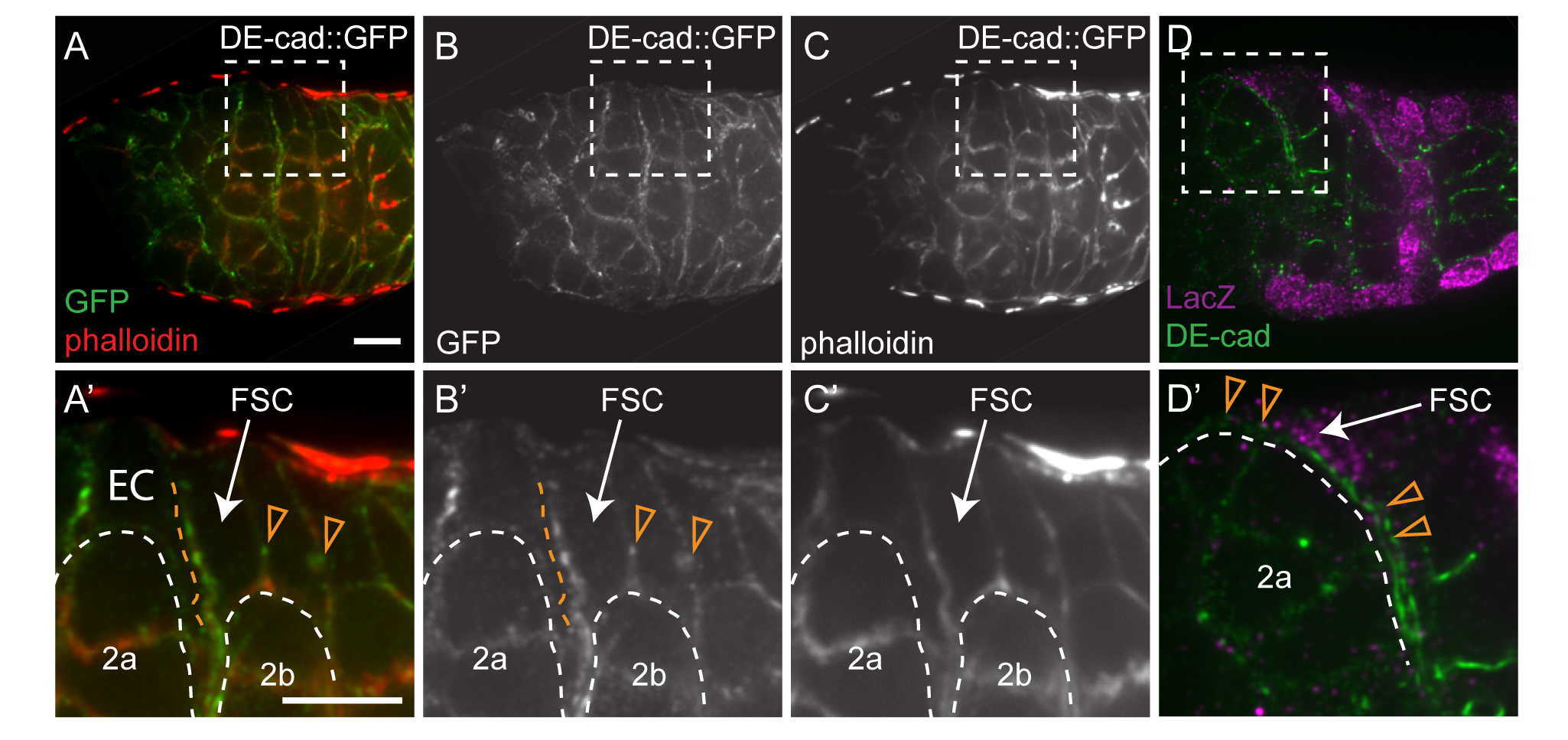

Supplement: Figure S2 — DE-cad is distributed broadly along the region of contact between FSCs and escort cells. A–C. DE-cad::GFP germarium stained for GFP (green, panel A) to label DE-cad, and phalloidin (red, panel A) to label cell membranes. The GFP channel and phalloidin channels are shown separately in B and C, respectively. The niche region (boxed in A–C) is magnified in A′–C′. A broad streak of DE-cad (orange dotted line in A′–B′) is visible on the anterior surface of the anterior most follicle cell, which is likely to be an FSC, whereas DE-cad is restricted to small puncta in the apical-lateral region of more posterior follicle cells (orange triangles). At this resolution, the surface of the escort cell (EC) that contacts the 2a cyst can be distinguished from the surface that contacts the FSC, revealing that the streak of DE-cad is between an FSC and EC. D. Germaria with a mature LacZ+ clone stained for DE-cad (green) and LacZ (purple). The FSC is identified as the anterior most LacZ+ cell in the clone. A broad streak of DE-cad is present on the anterior surface of the cell (orange triangles), which can be distinguished from the surface of the escort cell that contacts the 2a cyst. The niche region (boxed in D) is magnified in D′. Images were acquired using a Nikon spinning disc confocal microscope with a CFI Apo TIRF 100x lens (N.A.: 1.49). Anterior is to the left. Scale bar represents 5 µm. (TIF) [file pone.0101085.s002.tif]

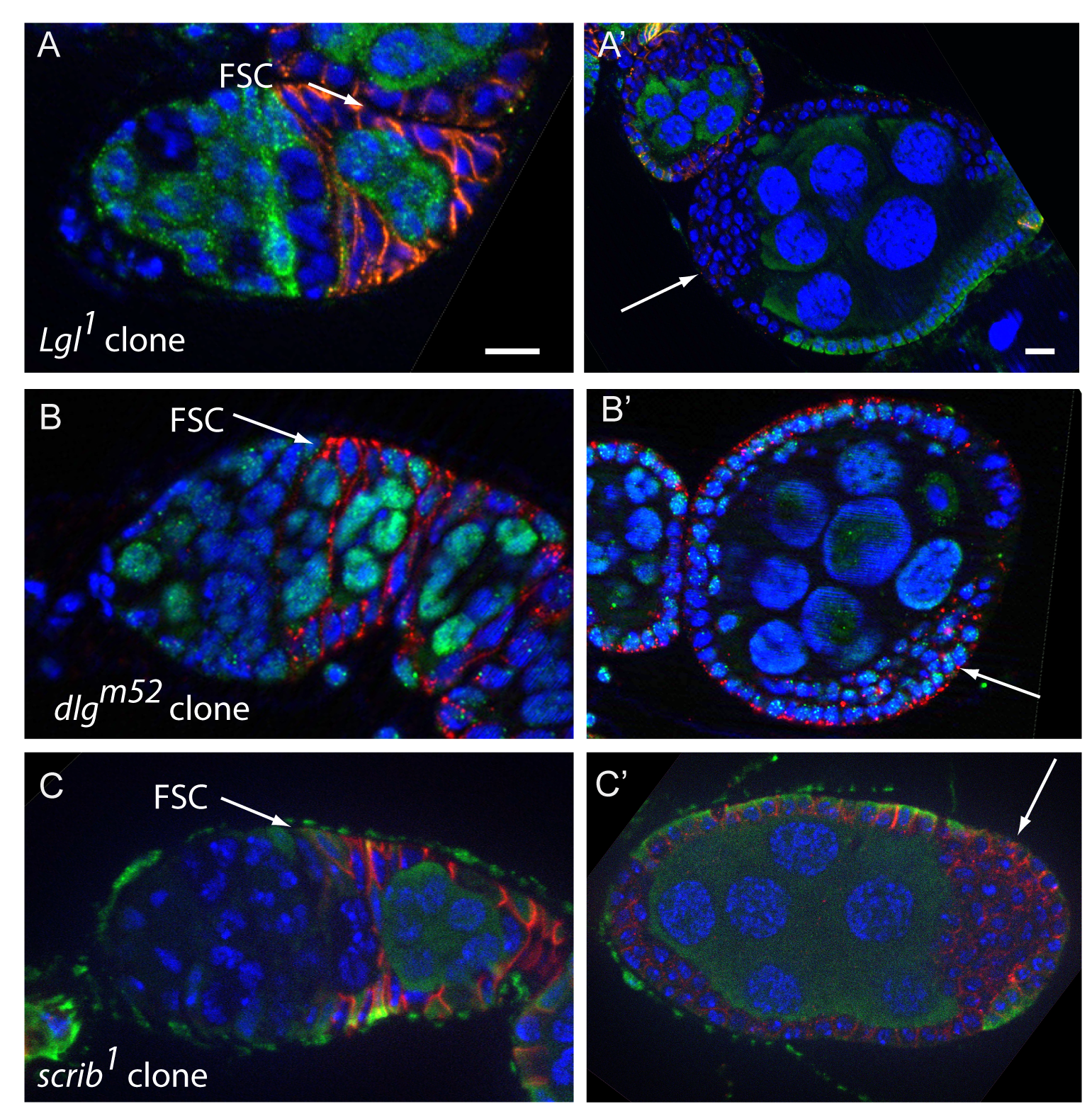

Supplement: Figure S3 — Dlg, Lgl, and Scrib mutations cause polarity defects but not hyperproliferation in the FSC niche region. A–C. Germaria with mature GFP- lgl1 (A), dlgm52/m52 (B), or scrib1 (C) FSC clones 14 days post heat shock stained for GFP (green), FasIII (red), and DAPI (blue). A′, B′ and C′ show follicles from the same ovarioles shown in A, B, and C, respectively. Thus, the follicle epithelium looks normal in the germarium, even at time points when significant neoplasia is observed in downstream follicles. Anterior is to the left. Scale bar represents 5 µm. (TIF) [file pone.0101085.s003.tif]

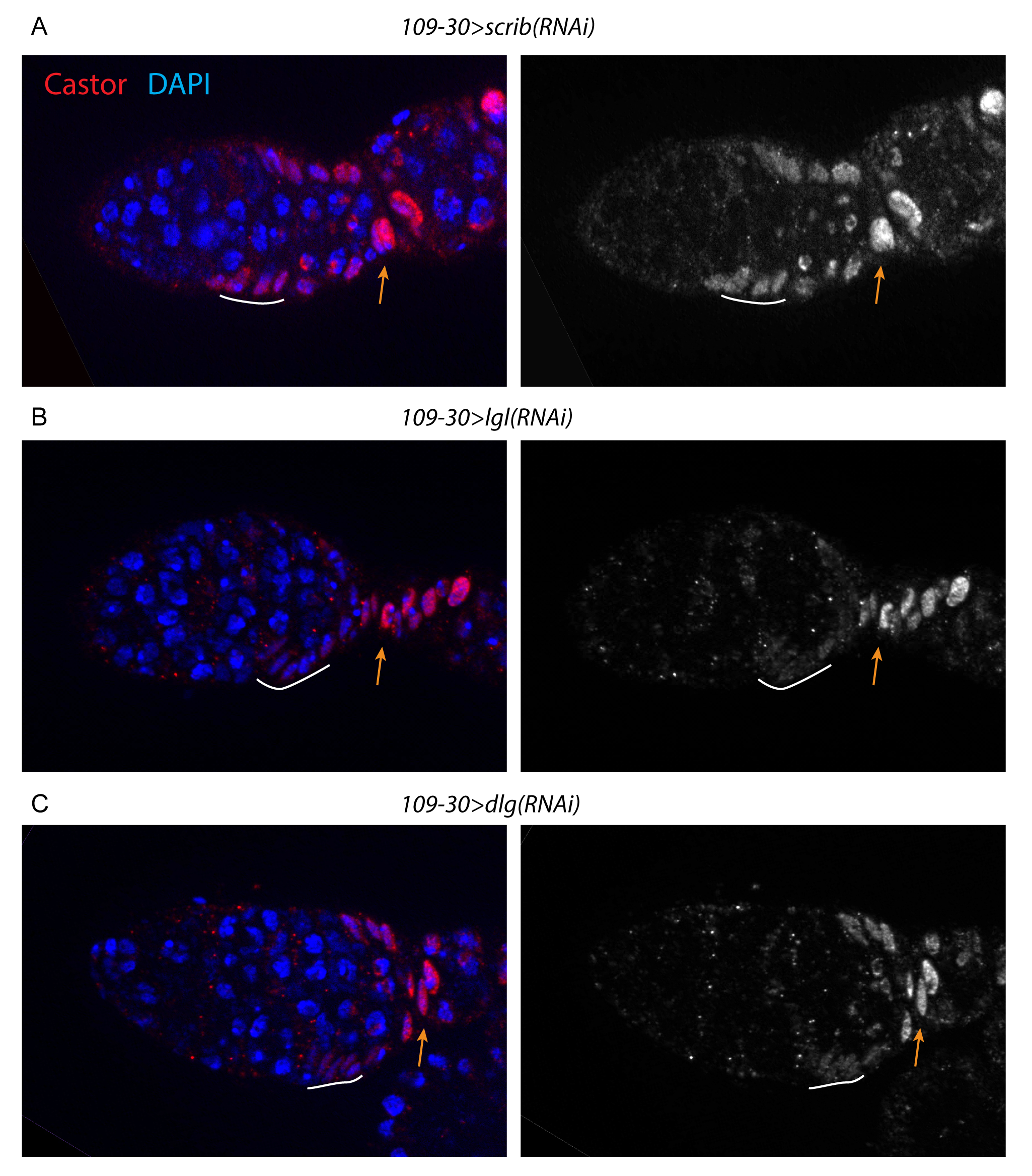

Supplement: Figure S4 — Knockdown of lgl or dlg but not scrib causes a decrease of cas expression in prefollicle cells. Germaria in which UAS-scribRNAi (A), UAS-lglRNAi (B), or UAS-dlgRNAi (C) expression is driven by 109-30-Gal4, a follicle cell driver, stained for cas (red) and DAPI (blue). cas expression levels in prefollicle cells (white lines) are substantially reduced in germaria expressing UAS-lglRNAi or UAS-dlgRNAi compared to prefollicle cells in germaria expressing UAS-scribRNAi. The consistently high expression of cas in stalk cells (orange arrows) served as a control for antibody staining and exposure times across samples. (TIF) [file pone.0101085.s004.tif]

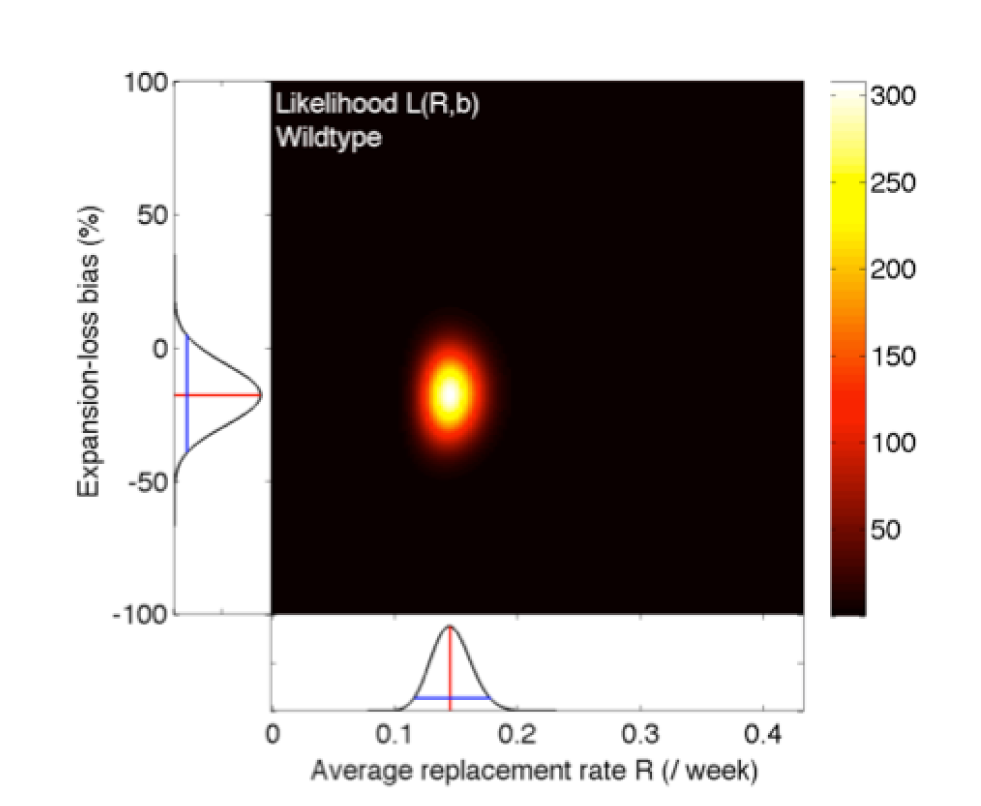

Supplement: Figure S5 — The Likelihood function. Parameter estimation for one data set (wild-type), showing the Likelihood function L(R,b), and the projected Likelihood for the two model parameters. The red lines indicate the maximum Likelihood estimates (MLE) of the parameters, and blue lines show the 95% confidence interval. (TIF) [file pone.0101085.s005.tif]
