# Supplementary material for: Basolateral Junction Proteins Regulate Competition for the Follicle Stem Cell Niche in the Drosophila Ovary
Source: PLoS One. 2014 Jul 3;9(7):e101085. doi: 10.1371/journal.pone.0101085 (PMC4084627; doi:10.1371/journal.pone.0101085)
Supplement: Table S1 — The maximum likelihood estimates of expansion rates (r+) and loss rates (r-) of mutant FSCs, normalized to wildtype. SE indicates the Standard Error. (DOCX) [file pone.0101085.s006.docx]

**Table S1:** Maximum likelihood estimates of FSC replacement rates, compared to wildtype

|  | Expansion rate (r_1_) | SE | Loss rate (r_2_) | SE |
| --- | --- | --- | --- | --- |
| Wildtype | 100% | N/A | 100% | N/A |
| lgl(1) | 386% | 120% | 19% | 21% |
| Dlg(m52) | 414% | 132% | 40% | 27% |
| Scrib(1) | 16% | 29% | 11% | 20% |
| Scrib(2) | 105% | 41% | 108% | 41% |
| Baz | 140% | 46% | 74% | 29% |
