# Supplementary material for: Basolateral Junction Proteins Regulate Competition for the Follicle Stem Cell Niche in the Drosophila Ovary
Source: PLoS One. 2014 Jul 3;9(7):e101085. doi: 10.1371/journal.pone.0101085 (PMC4084627; doi:10.1371/journal.pone.0101085)
Supplement: Table S2 — The maximum likelihood estimates (MLE) of the overall FSC replacement rate per week in germaria with FSC clones of the indicated genotypes. The standard errors and 95% confidence intervals are provided. (DOCX) [file pone.0101085.s007.docx]

**Table S2:** Maximum likelihood estimates of the overall FSC replacement rates in germaria with FSC clones of the indicated genotypes

|  | MLE (/week) | Std. Error (/week) | 95% CI (/week) |
| --- | --- | --- | --- |
| Wildtype | 0.146 | 0.019 | [0.116,0.182] |
| lgl(1) | 0.245 | 0.034 | [0.193,0.308] |
| Dlg(m52) | 0.280 | 0.035 | [0.224,0.350] |
| Scrib(1) | 0.024 | 0.025 | [0.004,0.060] |
| Scrib(2) | 0.156 | 0.021 | [0.123,0.196] |
| Baz | 0.148 | 0.019 | [0.119,0.179] |
