# Supplementary material for: Basolateral Junction Proteins Regulate Competition for the Follicle Stem Cell Niche in the Drosophila Ovary
Source: PLoS One. 2014 Jul 3;9(7):e101085. doi: 10.1371/journal.pone.0101085 (PMC4084627; doi:10.1371/journal.pone.0101085)
Supplement: Table S3 — The maximum likelihood estimates (MLE) for the competitive bias of marked FSCs. The standard errors and 95% confidence intervals are provided and the p-values are for the null hypothesis that competition is neutral (zero bias). (DOCX) [file pone.0101085.s008.docx]

**Table S3:** Maximum likelihood estimates for the competitive bias of marked FSCs, and *p*-values for the null hypothesis that competition is neutral (zero bias)

|  | MLE | Std. Error | 95% CI | *p*-value |
| --- | --- | --- | --- | --- |
| Wildtype | -19% | 14% | [-41%,5%] | 0.19 |
| lgl(1) | 87% | 16% | [63%,99%] | 3.0E-07 |
| Dlg(m52) | 75% | 13% | [53%,93%] | 6.9E-08 |
| Scrib(1) | -27% | 123% | [-94%,84%] | 1.000 |
| Scrib(2) | -20% | 14% | [-42%,5%] | 0.17 |
| Baz | 13% | 16% | [-9%,34%] | 0.42 |
