# Supplementary material for: Basolateral Junction Proteins Regulate Competition for the Follicle Stem Cell Niche in the Drosophila Ovary
Source: PLoS One. 2014 Jul 3;9(7):e101085. doi: 10.1371/journal.pone.0101085 (PMC4084627; doi:10.1371/journal.pone.0101085)
Supplement: Text S1 — Supplemental information describing the mathematical theory and modeling. Additional information provided about (1) the maximum likelihood parameter estimation; (2) null hypothesis testing; and (3) A generalized model for competitive bias in a multi-stem cell niche. (PDF) [file pone.0101085.s009.pdf]

THEORY SUPPLEMENT

1. Maximum Likelihood parameter estimation

To fit the model to the distribution of marked FSCs per ovary over multiple time points, we evaluated the Maximum Likelihood Estimators (MLE) (Wasserman, 2004) for the replacement rate *R*, and the bias *b* defined in the Methods section of the paper. Taking a non-informative (flat) prior for both parameters, the Likelihood function for a given parameter choice, *L*(*R*,*b*), is given by,

$$\ln[L(R,b)] = Z + \sum_i \left\{ n_{0,t_i} \ln\left[f_0(t_i|R,b)\right] + n_{1,t_i} \ln\left[f_1(t_i|R,b)\right] + n_{2,t_i} \ln\left[f_2(t_i|R,b)\right] \right\} \quad [\dagger]$$

where n_{k,t_i} are the empirical data giving the number of ovaries scored with *k*=0,1 or 2 GFP- marked FSCs at experimental time points t_i, and $f_k(t_i|R,b)$ are the theoretical frequencies for a given parameter choice *R* and *b*, shown in Eq. (*) in Methods. The quantity *Z* is a normalization constant ensuring $\int_{-1}^{1} db \int_0^{\infty} dR\, L(R,b) = 1$. Estimators for the parameters *R* and *b* can be evaluated as those maximizing *L*(*R*,*b*).

We evaluated the maximum Likelihood numerically using the MATLAB Optimization Toolbox subroutine `fminunc` (Tables S1-S3). To perform the maximization procedure, two additional implicit parameters must also be maximized, namely the (unknown) initial labeling frequencies $f_k(t_0|R,b)$ (*k*=1,2). Taking t_0=7 days, we find that these implicit parameters are very close to the empirical frequencies at 7 days post-labeling.
From Eq. [†] one also obtains the standard error and the 95% confidence intervals for *R, b* (Tables S2,S3). The standard error is evaluated by inverting the Hessian matrix of *L*(*R*,*b*). We obtained the Hessian matrix numerically in MATLAB (subroutine `fminunc`). To calculate the 95% confidence intervals for each of the parameters, we projected the Likelihood function for each parameter, defining $L_R(R) = \int_{-1}^{1} L(R,b)db$, and $L_b(b) = \int_0^{\infty} L(R,b)dR$, as the projected Likelihoods of *R* and *b* respectively. The 95% confidence intervals for parameter *X*∈{R,b} satisfy $q = \int_{-1}^{CI_{95}} L_X(x)dx$ with *q*=0.025 for the lower bound and q=0.975 for the upper bound. Figure S5 shows an example of the Likelihood function, the projected Likelihood functions, and fits with Maximum Likelihood parameters.

2. Null hypothesis testing

For each data set, we tested the significance level of the null hypothesis *b*=0, i.e. that competition is neutral. The *p*-values (Table S3) were calculated using the Likelihood Ratio Test (Wasserman, 2004). For this, we calculated the maximum Likelihood from Eq. [†], as before, but now assuming zero bias. The *p*-value was then evaluated as,

$$p = \mathrm{Prob}\left[\chi_q^2 > 2\log\frac{\sup_{(R,b)} L(R,b)}{\sup_{(R)} L(R,0)}\right],$$

where $\mathrm{Prob}\left[\chi_q^2 > X\right]$ is the chi-squared distribution with *q* degrees of freedom, and *q*=1 reflecting the number of degrees of freedom constrained in the null hypothesis relative to the unconstrained model.

3. A generalized model for competitive bias in a multi-stem cell niche

This section is intended for the more mathematically oriented reader, to extend the modeling framework used in the main part of the paper to a niche containing more than two stem cells. The key findings are summarized as follows: (1) we note that the same long-time clonal behavior emerges independently of the number of stem cells in the niche, and (2) the apparent bias *b* and replacement rate *R* fitted to an experiment through Eq. (*) of the Methods section are effective parameters (not true values of *R,b*), which depend on the number of stem cells and the geometry of the stem cell niche. (3) If more than two stem cells are present, the apparent average replacement rate *R* will depend on the

competitive bias, *b,* so that introduction of any bias (positive or negative) may appear to accelerate the rate of stem cell replacement. Expressions for *R*,*b,* are derived below for two stereotypical niche anatomies: one allowing nearest neighbor stem cell competition, and the other allowing all stem cells to compete with each other.

To fit the clonal dynamics arising from competition between stem cells in a confined niche of N_S stem cells, we study the probability distribution for the number of labeled stem cells per clone using a Master Equation for the fraction of clones $f_n(t)$ containing *n* stem cells at time *t* post-labeling,

$$\frac{1}{2R}\frac{df_n}{dt} = \frac{1+b}{2}(W_{n-1}f_{n-1} - W_n f_n) + \frac{1-b}{2}(W_{n+1}f_{n+1} - W_n f_n), \qquad (1)$$

for 1≤*n*≤N_S-1, solved subject to homogenous boundary conditions $\hat{f}_0(t) = \hat{f}_{N_S}(t) = 0$. The transition probability W_n is determined by the particular geometry of the niche: if the differentiation of a stem cell can be compensated by the division of any other stem cell in the niche, we have a logistic term $W_n = n(N_s - n)/N_s$, which describes the Moran Process (Moran, 1957); by contrast, if each stem cell competes with just two neighbors, as seen in the mouse intestinal crypt, we have (in a ring-like geometry) W_n = {1 for *n*=1,…,N_S-1; 0 for *n*=0}, (Klein et al., 2010; Lopez-Garcia et al., 2011; Snippert et al., 2011).

In solving Eq. (1) for different niche geometries, we are particularly interested in the long-time behavior of the clone size distribution, as it is found to follow a simple exponential relaxation of the type seen in the data. In particular, it will later become apparent that the fraction of niches that are either mosaic ($f_{\text{mos.}}$ - containing labeled and unlabeled cells), fully-labeled (f_{all}), or fully-unlabeled (f_{none}), asymptote to:

$$\lim_{t\to\infty} \quad f_{\text{mos.}}(t) = \sum_{n=1}^{N_S-1} f_n(t) = f_{\text{mos.}}^{(\text{eff.0})} e^{-2R_{\text{eff.}}t}$$

$$\lim_{t\to\infty} \quad f_{\text{all}}(t) = f_{N_S}(t) \quad = f_{\text{all}}^{(\text{eff.0})} + f_{\text{mos.}}^{(\text{eff.0})} \tfrac{1+b_{\text{eff}}}{2}[1 - e^{-R_{\text{eff.}}t}] \qquad (2)$$

$$\lim_{t\to\infty} \quad f_{\text{none}}(t) = f_0(t) \quad = \left[1 - f_{\text{all}}^{(\text{eff.0})} - f_{\text{mos.}}^{(\text{eff.0})}\right] + f_{\text{mos.}}^{(\text{eff.0})} \tfrac{1-b_{\text{eff}}}{2}[1 - e^{-R_{\text{eff.}}t}].$$

This dynamics has the same form as Eq. (*) (main text), and holds for any number of stem cells. Thus, when fitting clone fate data, the fractions $f_{\text{mos.}}^{(\text{eff.0})}$, $f_{\text{all}}^{(\text{eff.0})}$ appear to correspond to initial fraction of mosaic and fully labeled crypts in an experiment – but such a direct interpretation of the data is incorrect. Instead, $f_{\text{mos.}}^{(\text{eff.0})}$, $f_{\text{all}}^{(\text{eff.0})}$ depend on the history of clonal expansion and extinction at early times, and thus vary with the physiological parameters *R*, *b*, and N_S. Likewise, in general the effective parameters $R_{\text{eff.}}$, and b_{eff} both differ from the replacement rate *R* and bias *b*.

The goal below is to relate the effective parameters in Eq. (2) to the underlying parameters *R*, *b*, and N_S, and so to allow *R*, *b* to be inferred from relatively simple measurements. We begin with a case of nearest-neighbor stem cell competition, and then consider a case of competition between all stem cells in a niche.

3a. Stem cell competition with replacement of nearest-neighbors

This case has been recently explored in studies focusing on the mouse spermatogenesis and intestinal crypt turnover (Klein et al., 2010; Lopez-Garcia et al., 2011; Klein and Simons, 2011; Vermeulen et al., 2013; Snippert et al., 2014). We derive the previously published solution here for completeness, and then apply it to focus on the late-time behavior of the clonal dynamics.

General solution:

For this case, Eq. (1) becomes

$$\frac{1}{2R}\frac{df_n}{dt} = \frac{1+b}{2}(f_{n-1} - f_n) + \frac{1-b}{2}(f_{n+1} - f_n), \tag{3}$$

for 1≤n≤N_S, solved subject to homogenous boundary conditions $\hat{f}_0(t) = \hat{f}_{N_S}(t) = 0$.

To solve Eq. (3), we first identify the modal solutions $G_{k,n}(t) = \left(\frac{1+b}{1-b}\right)^{\frac{n}{2}} \sin\left(\frac{k\pi n}{N_S}\right) e^{-2\left[1-\sqrt{1-b^2}\cos\left(\frac{k\pi}{N_S}\right)\right]Rt}$ with 1≤k≤$N_S - 1$. The full solution has the form $f_n(t) = \sum_{k=1}^{N_S-1} a_k G_{k,n}(t)$ with pre-factors a_k set by the initial condition. Introducing an initial condition $f_n(0) = \delta_{n,n_0}$ corresponding to exactly n_0 stem cells contiguously labeled at *t*=0, the clone size distribution is found to be,

$$f_n(t) = \left(\frac{1+b}{1-b}\right)^{\frac{n-n_0}{2}} \sum_{k=1}^{N_S-1} x_{n,k}\, e^{-2R_k(N_S,b)t} \qquad \text{for } 1 \le k \le N_S - 1$$

$$f_0(t) = \sqrt{1-b^2}\left(\frac{1+b}{1-b}\right)^{-\frac{n_0}{2}} \sum_{k=1}^{N_S-1} \frac{R}{2R_k} x_{1,k}\left[1 - e^{-2R_k(N_S,b)t}\right] \tag{4}$$

$$f_{N_S}(t) = \sqrt{1-b^2}\left(\frac{1+b}{1-b}\right)^{\frac{N_S-n_0}{2}} \sum_{k=1}^{N_S-1} (-1)^{k+1}\frac{R}{2R_k} x_{1,k}\left[1 - e^{-2R_k(N_S,b)t}\right]$$

with $R_k(N_S,b) = \left[1 - \sqrt{1-b^2}\cos\left(\frac{k\pi}{N_S}\right)\right]R$, and $x_{n,k} = \frac{2}{N_S}\sin\left(\frac{k\pi n_0}{N_S}\right)\sin\left(\frac{k\pi n}{N_S}\right)$. Substituting in $N_S = 2$ recovers Eq. (*) given in the Methods section.

Late time dynamics:

At late times the lowest mode (*k*=1) in Eq. (4) dominates, so the clonal dynamics converge onto the same form as Eq. (2) for any value of N_S. From Eq. (4) we find the effective initial labeling fractions [see Eq. (2)] to be

$$\begin{aligned} f_{\text{mos.}}^{(\text{eff.0})} &= \sum_{n=1}^{N_S-1}\left[\left(\frac{1+b}{1-b}\right)^{\frac{n-n_0}{2}} x_{n,1} e^{-2R_1(N_S,b)t}\right] \\ &= \frac{2}{N_S}\sqrt{1-b^2}\left[\left(\frac{1+b}{1-b}\right)^{-\frac{n_0}{2}} + \left(\frac{1+b}{1-b}\right)^{\frac{N_S-n_0}{2}}\right]\frac{\sin\left(\frac{\pi n_0}{N_S}\right)\sin\left(\frac{\pi}{N_S}\right)}{1-\sqrt{1-b^2}\cos\left(\frac{\pi}{N_S}\right)} \quad , \\ f_{\text{all}}^{(\text{eff.0})} &= \frac{1-\left(\frac{1+b}{1-b}\right)^{n_0}}{1-\left(\frac{1+b}{1-b}\right)^{N_S}} - f_{\text{mos.}}^{(\text{eff.0})}. \end{aligned}$$

Similarly, the parameters $R_{\text{eff.}}$, b_{eff} apparent from the data do not give the true average replacement rate and bias. Instead, from Eq. (2) the effective quantities are related to the true parameters through the relations,

$$R_{\text{eff.}} = R_1(N_S,b) = \left[1 - \sqrt{1-b^2}\cos\left(\frac{\pi}{N_S}\right)\right]R,$$

$$\frac{1+b_{\text{eff.}}}{1-b_{\text{eff.}}} = \left(\frac{1+b}{1-b}\right)^{\frac{N_S}{2}}.$$

Note that $R_{\text{eff.}}$, $b_{\text{eff.}}$ are both independent of the initial distribution of labeled stem cells. One also sees a clear role for the niche size in establishing the outcome of competition, since a small bias *b* is amplified with increasing N_S.

3b. Stem cell competition with random replacement

The case where stem cells may be replaced by any other stem cells, irrespective of their position, corresponds to the Moran Process that has been widely studied in population genetics. Here, Eq. (1) hosts a logistic term $W_n = n(N_s - n)/N_s$.

Despite the long history of this model (Moran, 1958), no studies of the Moran process have been carried out specifically tracking clonal dynamics as relevant to the stem cell system. We are therefore not aware of a closed form solution that relates the experimentally-accessible parameters of Eq. (2), to the underlying parameters N_S, *R* and *b*. For the case of zero bias, an exact solution is provided by Houchmandzadeh and Vallade (Houchmandzadeh and Vallade, 2010); one finds $R_{\text{eff.}} = R/N_s$, $b_{\text{eff}} = 0$ for *b* = 0. For *b*≠0, Houchmandzadeh and Vallade pointed out that an exact solution to Eq. (1) leads to Heun's equation, for which no closed form solution is currently known. We provide below an approximation for the effective quantity $R_{\text{eff.}}$, and a heuristic expression for b_{eff}, although a full treatment of the problem is beyond the scope of this paper.

A well-known approximation to the Moran Process can be extracted from the seminal studies of Motoo Kimura in population genetics (Kimura, 1964), who solved Eq. (1) exactly in the limit of a large population ($N_s \gg 1$) and small bias ($N_s b \sim 1$). In this limit, Kimura showed that the eigenvalues R_k of Eq. (1) are related to those of the oblate spheroid wave equation through the relationship,

$$R_k = R[(N_S s)^2 - \lambda_{k,1}(N_S s)]$$

where the parameter *s* as originally defined by Kimura is simply related to our bias parameter as *s*=2*b* (see Appendix I of Houchmandzadeh and Vallade, 2010); the quantity $\lambda_{k,m}(x)$ is the eigenvalue of the oblate spheroid wave equation. Although there is no closed form expression for $\lambda_{1,1}$, the asymptotic behaviors are known,

$$R_{\text{eff}} = R_1 = \frac{R}{N_S}\begin{cases} 1 + \frac{(N_S b)^2}{10} - \mathcal{O}[(N_S b)^4] & \text{for } N_S b \ll 1 \\ N_S|b| - 1 - \frac{1}{N_S|b|} + \mathcal{O}[(N_S b)^{-2}] & \text{for } N_S b \gg 1 \end{cases}.$$

The case $N_S b \ll 1$ is reviewed in (Kimura, 1964), and the case $N_S b \gg 1$ is derived from the asymptotic expansion of $\lambda_{k,m}(x)$ in (Do-Nhat, 2001). Finally, the effective bias obtained by fitting Eq. (2) to the numerical solution of Eq. (1) shows excellent agreement with the case of nearest-neighbor replacement,

$$\frac{1+b_{\text{eff.}}}{1-b_{\text{eff.}}} = \left(\frac{1+b}{1-b}\right)^{\frac{N_S}{2}}.$$

Supplementary Theory references

Do-Nhat T (2001). Asymptotic expansions of the oblate spheroidal eigenvalues and wave functions for large parameter c. *Canadian J Phys* **79**, 813–831.

Houchmandzadeh B, Vallade M (2010). Alternative to the diffusion equation in population genetics. *Phys Rev E* **82**, 051913.

Kimura M (1964). Diffusion Models in Population Genetics. *J App Prob* **1**, 177–232.

Klein AM, Simons BD (2011). Universal patterns of stem cell fate in cycling adult tissues. *Development* **138**, 3103–3111.

Lopez-Garcia C, Klein AM, Simons BD, Winton DJ (2010). Intestinal stem cell replacement follows a pattern of neutral drift. *Science* **330**, 822–825.

Moran PAP (1958). Random Processes in Genetics. *Math Proc Camb Phil Soc*, **54**, 60–71.

Snippert HJ, Schepers AG, Van Es JH, Simons BD, Clevers H (2014). Biased competition between Lgr5 intestinal stem cells driven by oncogenic mutation induces clonal expansion. *EMBO Reports* **15**, 62-69

Vermeulen L, Morrissey E, van der Heijden M, Nicholson AM, Sottoriva A, Buczacki S, Kemp R, Tavaré S, Winton DJ (2013). Defining stem cell dynamics in models of intestinal tumor initiation. *Science* **342**, 995–998.
